# Stochastic seeding and environmental stressors as dual drivers of pioneer microbial colonization in newly formed basaltic lava tubes

**DOI:** 10.1186/s40793-026-00874-y

**Published:** 2026-03-20

**Authors:** Ana Z. Miller, Sara Gutierrez-Patricio, Fernando Gázquez, Alba Gomez-Arias, Javier Martínez-Martínez, Pedro Nolasco-Jiménez, Jorge R. Osman, Angel Fernández-Cortés, David Sanz-Mangas, Julio Castillo, Nicasio T. Jiménez-Morillo, Octavio Fernández-Lorenzo, Ana Pires, José M. Calaforra, Raúl Pérez-López, Beatriz Cubero, Nicoletta Fusi, Inés Galindo, Juana Vegas

**Affiliations:** 1 Instituto de Recursos Naturales y Agrobiología de Sevilla—Consejo Superior de Investigaciones Científicas (IRNAS-CSIC), Avenida Reina Mercedes 10, 41012 Seville, Spain; 2 HERCULES Laboratory, University of Évora, Largo Marquês de Marialva 8, 7000-809 Évora, Portugal; 3 Department of Biology and Geology, Universidad de Almería, Carretera de Sacramento S.N, La Cañada de San Urbano, 04120 Almería, Spain; 4 Andalusian Centre for Global Change—Hermelindo Castro (Engloba), Universidad de Almería, 04120 Almería, Spain; 5 Department of Chemistry, University of the Free State, 205 Nelson Mandela Dr, Bloemfontein, 9301 South Africa; 6 Instituto Geológico y Minero de España (IGME-CSIC), La Calera 1, Tres Cantos, 28760 Madrid, Spain; 7 Instituto de Geología Económica Aplicada (GEA), Universidad de Concepción, Concepción, Chile; 8 Instituto Geológico y Minero de España (IGME-CSIC), Unidad Territorial de Canarias, C/ Alonso Alvarado 43, 2A, 35003 Las Palmas de Gran Canaria, Spain; 9 Departamento de Ciencias Integradas, Universidad de Huelva, Avda. Fuerzas Armadas s.n, 21001 Huelva, Spain; 10 GE Tebexcorade—La Palma, Federación Canaria de Espeleología, La Palma, Spain; 11 INESCTEC—Institute for Systems and Computer Engineering, Technology and Science, Centre for Robotics and Autonomous Systems (CRAS|LSA), DEE, School of Engineering (ISEP), Polytechnic of Porto, 4200-072 Porto, Portugal; 12 Instituto Geológico y Minero de España (IGME-CSIC), Ríos Rosas 23, 28003 Madrid, Spain; 13 University of Milano-Bicocca, Piazza Della Scienza U4, 20126 Milan, Italy

**Keywords:** Volcanic caves, Sulfate-rich minerals, Geomicrobiology, Microbial succession, Tajogaite volcano

## Abstract

Lava tubes formed during the 2021 Tajogaite eruption on La Palma (Canary Islands, Spain) provide a rare opportunity to investigate the earliest stages of microbial colonization in a newly created subterranean volcanic environment. Although microbial communities in volcanic terrains have been widely studied, the first colonizers of newly formed lava tubes and their interactions with new mineral surfaces remain poorly understood. Here, we combined microscopy, mineralogy, stable isotope analysis, culture-dependent techniques, and 16S rRNA gene high-throughput sequencing to characterize mineral substrates, microenvironmental parameters and pioneer microbial communities 12–24 months after lava tube formation. Microbial assemblages were dominated by bacteria from the *Actinomycetota, Bacillota*, and *Pseudomonadota* phyla, together with archaea from the *Methanobacteriota* phylum. Organic debris from animals (notably seabirds and rodents) likely contributed to early microbial seeding and community development, as indicated by the detection of host-associated and opportunistic bacteria (e.g., *Staphylococcus*, *Sphingomonas*, *Pseudomonas*). Biofilm-like structures and extracellular polymeric substances were observed in association with sodium-rich sulfate and carbonate minerals, suggesting incipient microbe-mineral interactions during early mineral weathering. Archaeal-enriched communities adapted to highly saline and oligotrophic substrates (e.g., *Halostagnicola*, *Halonotius*, and *Halorubrum*) in some of these nascent tubes further support environmental filtering by microhabitat geochemistry and mineralogical context during early community assembly. Our findings indicate that early microbial colonization is initiated by stochastic seeding processes (via aerosols, animals, or atmospheric inputs), whereas environmental stressors related to volcanic degassing, ventilation, and extreme geochemical conditions act as strong deterministic filters that shape the final community structure in newly formed basaltic lava tubes, as reflected by the significant role of *δ*^13^C–CO_2_.

**Graphical abstract:**

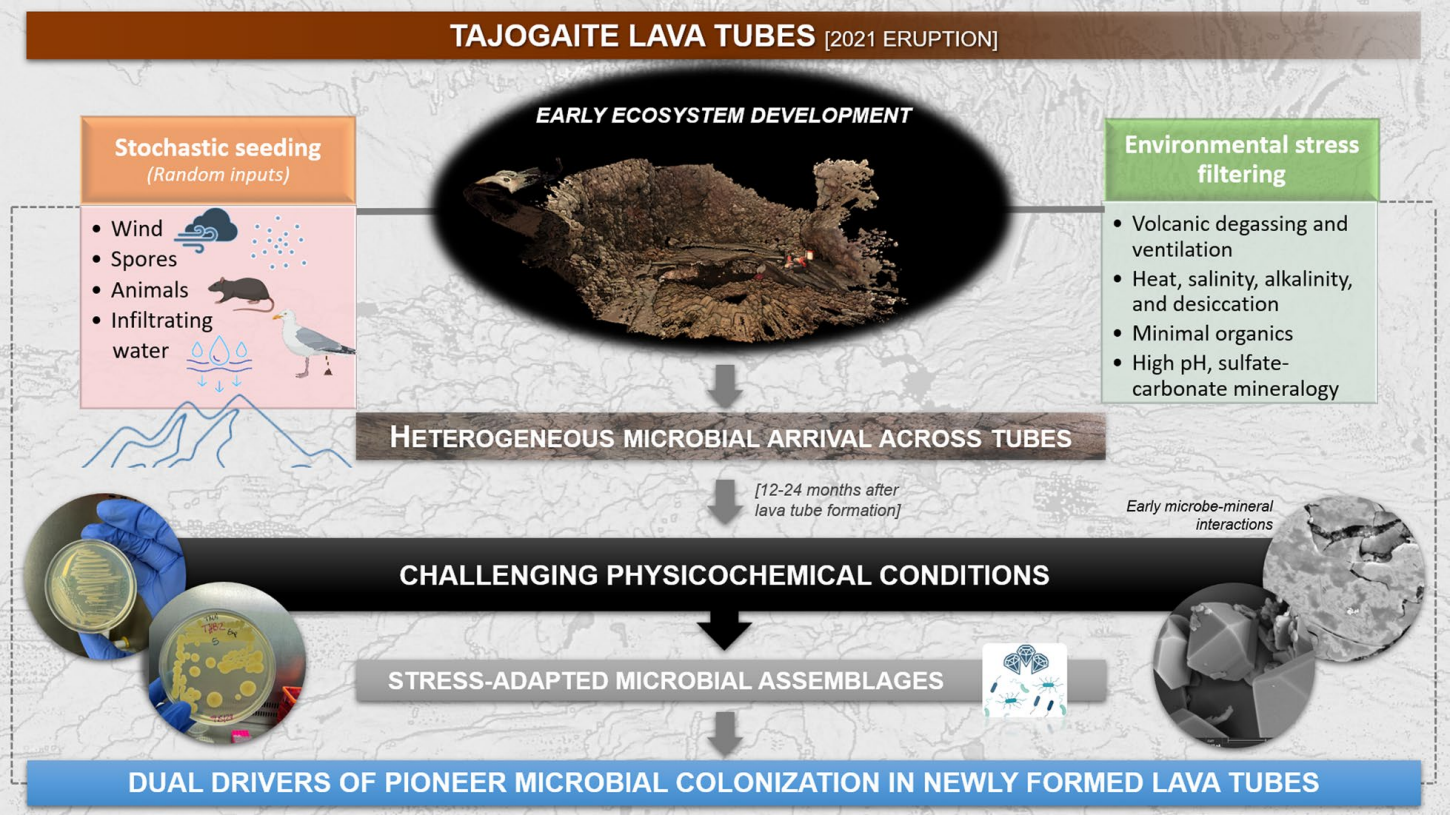

## Introduction

Volcanic eruptions are among the most transformative forces on Earth, rapidly reshaping landscapes and creating new ecosystems from molten rock. In 2021, the Tajogaite eruption on La Palma Island (Canary Islands, Spain) exemplifies this geological dynamism. In a matter of weeks, tons of lava and tephra redefined the island’s topography and gave rise to a pristine and sterile volcanic terrain [1].

Biological seeding of newly emplaced lava fields can occur through aeolian inputs, rain droplets, rainwater streams, animal activity, and anthropogenic contamination [2]. Pioneer photoautotrophic microorganisms like green microalgae and cyanobacteria, as well as chemolithoautotrophic bacteria, play a crucial role in establishing initial communities on lava flows [3]. These organisms thrive by utilizing minerals from volcanic rocks and energy from sunlight or chemical processes, making them the primary producers in newly formed lava fields [2, 4]. In Iceland, thermophiles and psychrophiles, belonging to *Acidobacteriota*, *Actinomycetota*, and *Pseudomonadota* phyla, have been commonly reported on freshly cooled lava flows, reflecting adaptation to temperature gradients and geothermal activity [4].

Beneath the new and still-hot surface, created by low-viscosity lava, molten rock continues to flow underground, forming lava tubes. Once the eruption is over and the lava stops flowing, lava tubes become accessible through different types of entrances, either formed while the lava was flowing or once the tube has drained, such as roof collapses or skylights [5, 6]. These underground tunnels present a unique and extreme environment for microbial colonization, sustained by surface-derived organic inputs delivered by dripping water, aerosols, dust deposition, or in-situ CO_2_ fixation [7]. Following the establishment of chemoautotrophic primary producers, lava tubes rapidly promote the growth of diverse microbial communities, contributing to biogeochemical processes and rock bioweathering [4, 8]. In lava tubes from Hawai'i (USA), Iceland, the Azores (Portugal), and the Canary Islands (Spain), microbial communities show high resilience and metabolic versatility, commonly dominated by *Actinomycetota* and *Pseudomonadota* [9, 10, 12,11], . Northup et al. [10] and Prescott et al. [13] reported microbial metabolisms largely driven by sulfates, sulfides, or reduced iron as electron donors in lava tubes from Hawai'i Volcanoes National Park. In the Canary Islands, the occurrence of metabolically active *Actinomycetota* in yellow mats from La Palma and Tenerife underscores their specialization and potential involvement in bioleaching and rock weathering [14, 101]. Beyond their roles in nutrient cycling and mineral transformation, microorganisms dwelling in lava tubes are increasingly recognized as a source of biotechnologically relevant metabolites, as cave-derived isolates have yielded bioactive compounds with antimicrobial and antitumoral activities [113, 114]. Moreover, subsurface microbial life can mediate mineral transformation, metal mobilization, and bioleaching, which are key processes in biomining and potentially applicable to in situ resource utilization under extreme conditions [16–18]. At the same time, the growing evidence for lava tubes on Mars [6], together with the environmental gradients and subsurface isolation that characterize terrestrial lava tubes, makethese systems powerful analogues to test strategies for detecting microbial life and assessing habitability in extraterrestrial subsurface settings [10, 19, 22, 105].

Nascent lava tube habitats, with their unique combination of energy constraints, elevated temperatures, and stable microclimates, therefore provide a unique opportunity to document microbial colonization and early ecological succession from their very inception. Yet, despite their ecological and applied significance, early-stage lava tube ecosystems remain unexplored.

 High-temporal-resolution surveys of newly formed lava flows in Iceland show that microbial communities can establish rapidly but variably, with the relative influence of deterministic processes (environmental filtering and biotic interactions) and stochastic processes (dispersal, ecological drift, and historical contingency) shifting over time [23]. In lava tubes, steep physicochemical gradients (e.g., temperature, moisture, pH, and mineralogical composition) may impose strong environmental filtering, while connectivity to external sources can modulate dispersal and early colonization dynamics.

The Tajogaite eruption in La Palma, which created an extensive network of newly formed lava tubes, opens a rare window into the earliest stages of subsurface ecosystem development, providing an exceptional opportunity to disentangle the mechanisms driving early succession in oligotrophic, mineral-dominated environments. Here, we present the first geomicrobiological investigation of the newly formed lava tubes of the Tajogaite Volcano, characterizing their mineral substrates, microenvironmental conditions, and pioneering microbial communities. By integrating geochemical and mineralogical data with microbial community analyses, we address two key questions: What are the drivers of early microbial colonization on newly formed lava tubes? How do microorganisms interact with fresh mineral surfaces?

## Methods and materials

### Geological setting

La Palma is an oceanic island located in an intraplate tectonic setting in the NW of the Canary Islands. With a maximum age of ~ 4 million years (Ma), it is the second youngest island of the archipelago and has developed through six major geological phases [15, 24, 29, 80, 98, 100, 102]: (1) Early submarine stages of Pliocene age (< 4 Ma); (2) Intense magmatic and dike intrusions that triggered low-grade metamorphism and uplifted the primitive seamount to altitudes of up to 1500 m asl (~ 2 Ma); (3) Two main superimposed stratovolcanoes in the northern part of the island: (i) Garafía: 1.7–1.2 Ma, and (ii) Taburiente: 1.1–0.4 Ma; (4) a series of massive gravitational landslides that reshaped the island, including Cumbre Nueva (0.4–0.5 Ma), Playa de la Veta (0.8–1.0 Ma), and Santa Cruz (1 Ma); (5) construction of the Bejenado volcanic edifice (0.6–0.5 Ma), which partially filled the primitive Taburiente caldera, forming a lake; (6) development of the Cumbre Vieja Ridge, characterized by aligned volcanic fissures and minor vents along a north–south trending ridge, with activity spanning the last 123,000 years and including eight historical Strombolian eruptions (from the fifteenth century to the present). During this last phase, a deeply incised fluvial network emerged, shaped by glacial/interglacial cycles, leading to the opening of the Las Angustias Gorge and the formation of marine platforms around Cumbre Nueva.

After five decades of quiescence, the most recent volcanic eruption on La Palma commenced on 19 September 2021 on the western flank of the Cumbre Vieja Ridge [30]. This monogenetic Strombolian eruption, later named Tajogaite, was characterized by fissural activity with intermittent phreatomagmatic pulses that conferred a hybrid eruptive nature [97]. The main eruptive fissure opened along an NW–SE-oriented fracture, structurally influenced by the previous fracture network [108].

**Fig. 1:**
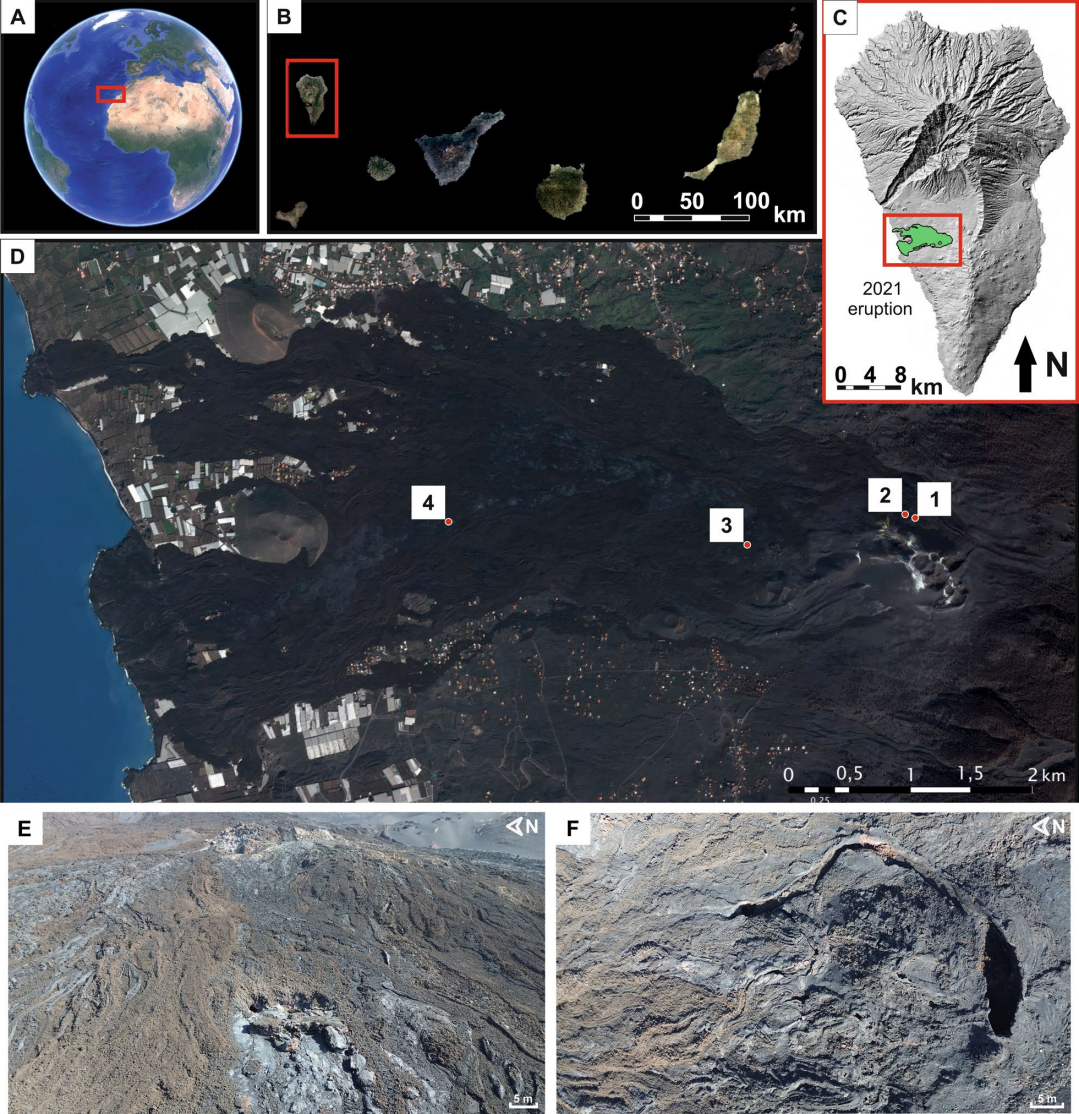
Location of the study sites. **A** Google Earth © Online image with location of the Canary Islands. Data: SIO, NOAA, U.S. Navy, NGA, GEBCO Image Landsat/Copernicus/IBCA/U.S. Geological Survey – Google Earth;** B** Photogrammetric flight GSD 2020–2021 image of the Canary Islands. Data: *Cartográfica de Canarias* S.A (GRAFCAN), *Gobierno de Canarias*—IDE Canarias. **C** Hillshade map 2022 of La Palma Island with 2021 Tajogaite eruption (green colour) on the west side of the island. Data: *Cartográfica de Canarias* S.A (GRAFCAN), *Gobierno de Canarias*—IDE Canarias; **D** 2022 orthophoto from Pléiades Satellite CNES (2021) of the area affected by the Tajogaite eruption with location of the lava tubes studied in this work: 1: *Sima Hornitos* Cave (TSH); 2: *Canal Hornito Bonito* Lava Tube (THB); 3: *Tubo Rojo* (TR); and 4: *Shatter Ring*-1 (TDS1). Data: Distribution Airbus DS (GRAFCAN), *Gobierno de Canarias*; **E,F** Drone images from the surface of the Tajogaite lava field four years after the eruption, characterized by bare basaltic flows and tephra

Over the course of 85 days, the Tajogaite eruption produced a sustained release of volcanic gases, tephra, and extensive lava flows, covering approximately 1200 ha, including both rural lands and several populated areas, such as Todoque, Los Campitos, La Laguna, Alcalá, and El Paraíso (Fig. 1A–D). These lava flows reached the coast and generated two lava deltas with a combined emerged area of 48 ha (plus 21 ha submarine). The eruption was classified as Volcanic Explosivity Index (VEI) 3 event [106].

The eruptive volume exceeded 217 Mm^3^ of lava [31], discharged from a N130°E-aligned main fissure approximately 0.5 km long, comprising more than a dozen emission centers. The resulting volcanic cone reached a height of ~ 200 m above the pre-eruptive terrain, with a basal width of 700 m and an elliptical summit crater measuring 172 × 106 m. The lava field reached a maximum thickness of 70 m and extended up to 6.5 km in length. Petrological analyses identified the emitted lavas as tephrite-basanite in composition [32], predominantly of the a ‘ā type, with minor pāhoehoe flows in the waning stages of the eruption. The tephra blanket, composed of blocks, bombs, lapilli, and ash, ranged from several meters thick near the vent to millimetric layers in distal zones, with ashfall recorded across the entire island and even in other western Canary Islands during the eruption’s most explosive phases [33].

An extensive and complex network of lava tubes formed concurrently with the emplacement of lava flows and the growth of the main cone. To date, nearly 30 entrances have been mapped, with several individual tubes extending over 3 km in length [109]. These tubes are fed by a main lava conduit descending the western slope of the cone, where deep skylights reveal at least two superimposed levels, each stretching approximately 1 km. Many of these newly formed tubes remain under exploration due to persistently high internal temperatures. They exhibit an extraordinary variety of lava features, including complex braided mazes, multi-level development, lava falls, and surface varnishes with unusual colorations. The internal height of the tubes varies from more than 25 m in the inaccessible skylights near the summit cone, to more typical dimensions of 4–6 m, tapering to narrow conduits under 0.5 m. This morphological diversity is largely attributed to multiple processes, including roof collapses, lava thickening, internal lava upwelling that produced surface overflows before re-entering the main conduit, and slope-induced changes that generated geomorphological features such as shatter rings [34].

### Studied sites and sample collection

To monitor the initial stages of microbial colonization in newly formed lava tubes from the 2021 Tajogaite eruption on La Palma Island, we conducted three comprehensive sampling campaigns within 12, 18 and 24 months after the eruption (January 2023–2024). These campaigns focused on four lava tubes that became accessible as temperatures decreased, ensuring safety conditions to access these new volcanic cavities, with the necessary permissions from the authorities responsible for managing access to the exclusion area.

The newly formed lava tubes are located within the Tajogaite lava flow field (Fig. 1C,D), which emplaced urban areas, agricultural soils and pine forest. The terrain surrounding the new caves consists almost entirely of bare basaltic flows and tephra, with no developed soil, carbonized wood, or vegetation owing to the recent emplacement of lava locally exceeding 60 m in thickness [97]; Fig. 1E,F). During the 2024 sampling campaign we observed fresh bird guano (seabirds and pigeons, and kestrels), feathers, nesting material, and spider webs at several tube entrances (Fig. S1).

**Fig. 2:**
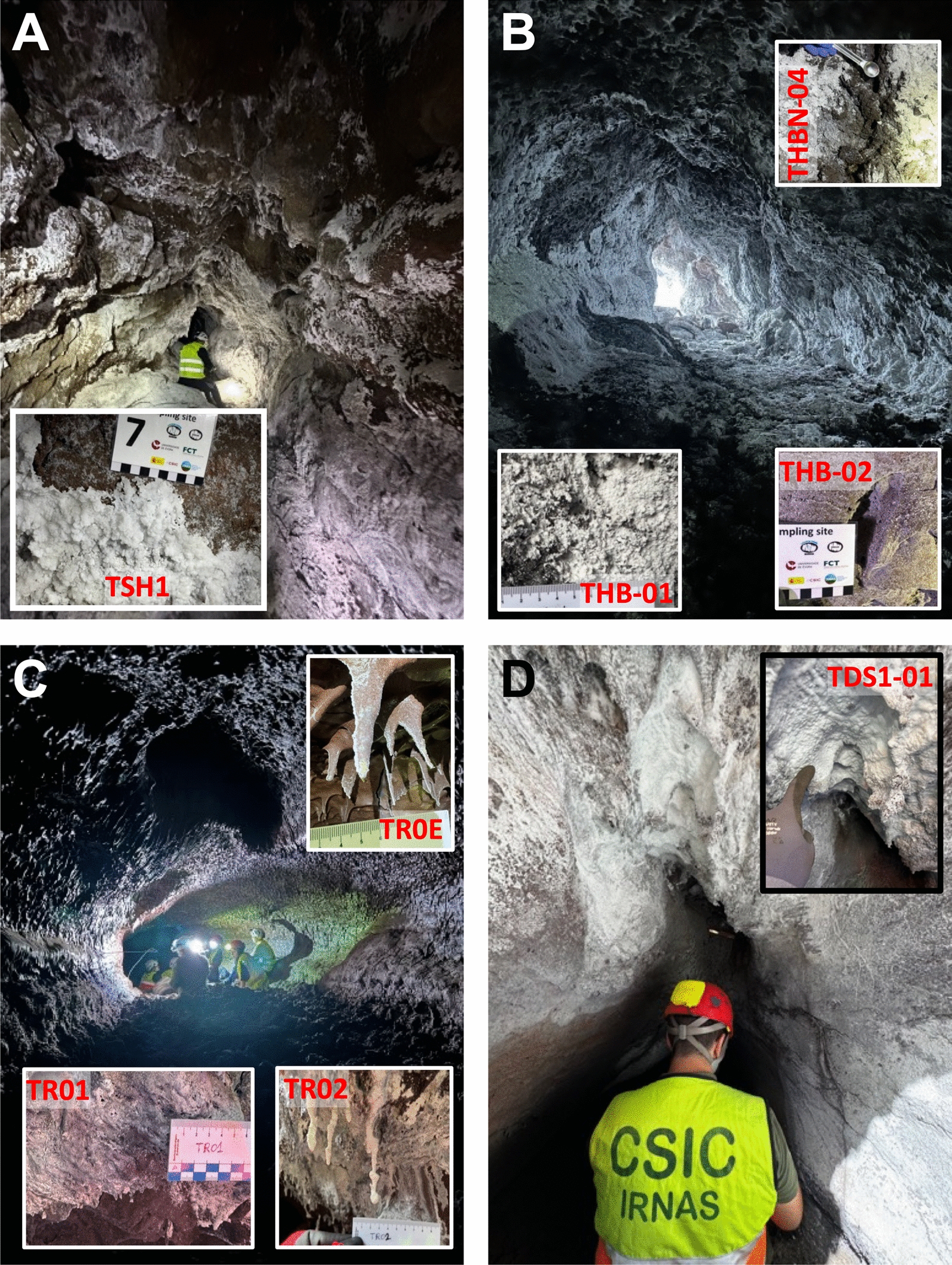
Field images of the main features and morphology of the newly formed lava tubes of the Tajogaite Volcano in La Palma and corresponding sampling points. **A** *Sima Hornitos* (TSH); **B** *Canal Hornito Bonito* Lava Tube (THB); **C** *Tubo Rojo* (TR), and **D** *Shatter Ring-1* (TDS)

***Sima Hornitos*** (TSH) is a multilevel lava cave located in the northern and highest part of the lava field (Fig. 1D). It comprises a hollow volcanic dyke or fissure with two partially superimposed levels. The upper conduit extends 22 m and is characterized by *hornitos* (small rootless spatter cones formed by the extrusion of pressurized lava and gas through fractures in the lava crust) on the roof, spiny walls, and lava tube boxwork (Fig. 2A). The walls are coated with newly formed white soft powdery crusts, and translucent stalactites measuring 12–15 cm in length (Table S1). This cavity is encoded as S_Sh_01 in the Tajogaite caves catalogue developed by IGME-CSIC.

***Canal Hornito Bonito*** Lava Tube (THB), also located in the northern part of the lava field (Fig. 1D), is 47 m long, with an entrance situated in the lower end of a lava channel. The floor is composed of aa clinker, and the ceiling is adorned with lava stalactites (Fig. 2B). The walls display flow line features and sections with stretched lava projections, coated with white powdery deposits (Table S1). The *Canal Hornito Bonito* Lava Tube is encoded as S_Hb_02 in the Tajogaite caves catalogue developed by IGME-CSIC.

***Tubo Rojo*** (TR) is a highly complex lava tube with multiple branches located about 1 km downslope from the cone (Fig. 1D). It provides access to the Paraíso-Todoque lava-tube system, whose total length likely exceeds 3 km, although most passages remain unexplored due to persistent heat (100–200 ºC). The sampled area in TR spans approximately 72 m from the entrance. The most significant feature of this tube is the complete vitreous reddish skin covering the ceiling and walls (Fig. 2C). Occasional whitish powdery deposits were found to predominantly grow on one side of lava stalactites, opposite to the cave entrance (sample ID TR0E, Table S1), suggesting a connection with the main airflow direction in the lava tube. The lower entrance of this tube is encoded as S_P-T_02 in the Tajogaite catalogue by IGME-CSIC.

***Shatter Ring-1*** (TDS1) is a sub-crustal lava tube that rapidly narrows to a crawlway, located near the newly reconstructed LP-211 road in the south-central sector of the Tajogaite lava field (Fig. 1D). The access to this cavity is via an open, roofless segment where samples were collected (Fig. 2D).

To ensure safe access to these newly formed cavities, we monitored volcanic gas presence and temperature distribution inside each lava tube during every field campaign. Volcanic gas inside the caves was monitored using Industrial Scientific iBRID MX6 and Ventis Pro gas detectors for O_2_, CO, CO_2_, HCl, H_2_S, SO_2_ and CH_4_. Thermal mapping was carried out using a DJI Mavic 2 Enterprise Advance drone equipped with a handheld thermal infrared camera (640 × 512 at 30 Hz resolution sensor, 8–14 µm spectral band), complemented by in situ measurements with a TENMA 72–7715 digital handheld thermometer fitted with Type K thermocouple probes for both surface and internal rock temperatures. The latter was performed in 90 cm deep holes, 12 mm diameter, drilled in the walls using a Bosch GBH 36 VF-Li Professional battery-powered drill. These field measurements were essential to verify that temperatures had decreased sufficiently to allow safe entry and to confirm that the lava substrate temperatures continue to decrease even several years after the eruption (Table 1; Fig. S2 and S3).

**Table 1:** CO_2_ concentration, *δ*^13^C of CO_2_, temperature (T) and relative humidity (RH) in the air of the lava tubes and the exterior atmosphere, measured two years after the eruption

Type	Location	CO_2_ (ppm)	*δ*^13^C-CO_2_ (‰)	T (^o^C)	RH (%)
Exterior	*Tubo Rojo*	440 (± 0.21)	−10.33 (± 0.03)	16.8	68
	*Canal Hornito Bonito*	464 (± 0.35)	−11.80 (± 0.03)		
Interior of lava tube	*Sima Hornitos*	541 (± 0.70)	−3.06 (± 0.08)	21.0	72
	*Canal Hornito Bonito* (end zone)	502 (± 0.38)	−14.54 (± 0.33)	18.3	65
	*Tubo Rojo* (middle zone)	529 (± 0.34)	−13.34 (± 0.03)	28.9	36
	*Tubo Rojo* (end zone)	527 (± 0.28)	−1.10 (± 0.03)	> 55	24

A total of 35 samples, predominantly consisting of whitish mineral deposits, were collected from the walls, floors, and ceilings of four lava tubes for mineralogical, geochemical, and microbiological characterization (Table S1). All investigated lava tube sections (*Sima Hornitos*, *Canal Hornito Bonito*, *Tubo Rojo*, and *Shatter Ring-1*) are partially collapsed structures (typically only a few to several metres long), allowing natural light to penetrate throughout their length. Although no instrumental measurements of light intensity were performed, qualitative observations indicated a clear decreasing gradient of illumination from the entrance toward the interior. Samples TR0E, from *Tubo Rojo* Lava Tube, and TSH1, from *Sima Hornitos* were collected in the brightest entrance-proximal areas, whereas the remaining samples were taken in progressively dimmer sections (Fig. 2; Table S1).

For microbiological analysis, mineral-coated surfaces were sampled by scraping the mineral deposits with individually wrapped sterile disposable scalpels or by swabbing the surfaces with sterile cotton swabs to collect any loosely attached biomass. All sampling procedures were performed wearing face masks, and nitrile gloves to minimize contamination. All microbiology samples were placed into sterile Whirl–Pak sampling bags. For mineralogical and geochemical analyses, samples were collected with spatulas sterilized in the field with 70% ethanol and stored in sterile 50-ml Falcon tubes. All samples were then transported to the laboratory under refrigerated conditions (~ 4 °C) and processed upon arrival.

This study focuses specifically on eight sampling sites from four lava tubes where DNA was successfully extracted and bacterial isolates were obtained (Fig. 2; Table S1).

Air samples were collected in 2024 at the investigated lava tubes (*n* = 6) and outside (*n* = 2), near the cave entrances. Air temperature, relative humidity, and CO_2_ concentrations were measured in-situ using an XP200 device (Lufft), equipped with an external temperature and relative humidity probe (8120.TFF, Lufft) with an accuracy at 20 °C of ± 0.1 °C and ± 3% above 90%, respectively, and a dual wavelength NDIR CO_2_ probe (EE871, E + E Elektronik) with a measurement range of 0–10,000 ppm, an accuracy (at 25 °C and 1013 mbar) < ± 100 ppm (+ 5% from the measured value). A portable air compressor was operated at 0.4 L·min^−1^ to collect the air mixture from each lava tube. Air samples were stored in 1L Ritter bags with lock valves.

Temperature within *Canal Hornito Bonito* Lava Tube and *Sima Hornitos* was monitored during several months using a Corentium Pro (Airthings) data logger equipped with integrated environmental sensors. The temperature sensor had a resolution of 0.33°C and an accuracy of ± 1°C. Relative humidity was measured with a resolution of 0.5% and an accuracy of ± 4.5%.

### Cave atmosphere characterization and stable isotope analysis (***δ***^13^C) of CO_2_

Air samples were analysed within 48 h of sampling to determine CO_2_ concentration and the *δ*^13^C value, using a CRDS system (G2201-i Analyzer, Picarro, USA) at the Stable Isotope Laboratory of the University of Almeria. Three internal standards with certified gas mixtures and known concentrations of CO_2_ (7000 ppm, 400 ppm, and zero CO_2_), supplied by Abello Linde-Spain, were used to calibrate the CO_2_ concentration values of the air samples. The proper functioning and performance specifications of the CRDS analyser for *δ*^13^C-CO_2_ analyses were initially checked and calibrated using several standards supplied by the USGS/Reston Stable Isotope Laboratory (USGS-40 and USGS-41a) and IAEA (IAEA-603 and NBS-18), utilizing a combustion module (Costech, USA) coupled to the CRDS analyser (CM-CRDS system). The *δ*^13^C-CO_2_ analyses of the air samples were calibrated against CO_2_ obtained by the CM-CRDS system for the following internal secondary standards: NaHCO_3_, sugarcane, acetanilide, and urea, covering a *δ*^13^C calibration range from approximately −4 to −49‰. The stable carbon isotope compositions of CO_2_ were expressed as *δ*^13^C relative to the standard Vienna Pee Dee Belemnite (VPDB). CO_2_ isotopologue measurements achieved a precision of 200 ppb (± 0.05) and 10 ppb (± 0.05) for ^12^CO_2_ and ^13^CO_2_, respectively, resulting in a precision greater than 0.16 for *δ*^13^C-CO_2_.

### Morphological and mineralogical characterization

On-site observations were carried out using a portable handheld digital microscope (DinoLite WF4115ZTL) at up to 140 × magnification. This allowed observing mineral habits, textures and morphologies without the need for sampling or transport, consequently avoiding the breakage of structures.

Representative samples of whitish minerals collected in the lava tubes were examined using a FEI INSPECT™ scanning electron microscope (SEM) at *Museo Nacional de Ciencias Naturales* (MNCN-CSIC) and *Instituto Geológico y Minero de España* (IGME-CSIC), using a JEOL JSM-6010 LA PLUS SEM. Uncoated samples were observed and imaged in low-vacuum mode (40 Pa) with accelerating voltages of 15 kV, 20 kV, and 30 kV at a working distance of 9–11 mm. Photomicrographs were taken in shadow-backscattered electron imaging mode (BSE). Qualitative and semi-quantitative Energy Dispersive X-ray Spectroscopy analysis (EDS) was performed under the same low-vacuum (LV) conditions using OXFORD INSTRUMENTS analytical-INCA.

Field emission scanning electron microscopy (FESEM) was also conducted to detect microbial cells and biogenic mineral structures, using a high-resolution FEI Teneo FESEM (FEI Company, Eindhoven, The Netherlands) equipped with an Oxford X-ray energy dispersive spectroscopy (EDS) detector. Air-dried bulk samples were directly mounted on sample stubs and sputter-coated with platinum. The instrument was set up between 5 and 15 kV and 0.4–1.6 nA using the detector ETD in SE mode, detector T1 in mode A + B and detector ABS in mode All, WD 9.9–10.1 mm, magnification range 800–24,000 × and HFW: 8.63 –259 μm. The EDS analyses were performed at 15 kV, magnification 1200–6000 x, Takeoff 33.2–35.3, live time 30 s, amp time 0.96 μs and 127.7 eV of resolution.

The most developed precipitates (stalactites and crusts) were analyzed using a high-resolution X-ray micro-computed tomography (micro-CT) to investigate the presence of internal textures and/or structures. The micro-CT system used is a BIR Actis 130/150 with a polychromatic X-ray generator, with an energy of 100 keV/80 mA to scan the samples. 3D images were reconstructed both with Actis and Avizo-Fire software. The dimensions of the voxel, corresponding to the resolution of the images, were 10 × 10 × 10 µm.

Samples for powder X-ray diffraction (XRD) analyses (~ 100 mg) were ground and dried at 40 °C overnight. Analyses were conducted at the Technical Service Area of the University of Almería (Spain) using an X-ray diffractometer D8 ADVANCE Model DAVINCI, with a Cu anode (CuKα, λ = 0.154 nm) and a graphite monochromator. A Ni filter and Al sample holders were utilized. The tension and current produced by the generator were 40 kV and 30 mA, respectively, for all analyses. The analysis used the 2θ scanning method, with 0.400 s per step and within the angular limits of 5–80°. Mineralogical determination used the PDF-2 (Powder Diffraction Files) database.

### Culture-dependent techniques

Subsamples for microbiology were resuspended in sterile 0.85% (w/v) NaCl solution and subsequently seeded on nutrient agar (NA, Difco), tryptone-soy agar with magnesium and sodium (TSBANaMg), agar marine medium, *Nitrososphaera* medium (DSMZ #1630) and halophilic Archaea medium (DSMZ #1184). All samples were incubated at 30 °C for 7 weeks. Microbial colonies were then selected according to their morphological characteristics for isolation and further taxonomic identification by 16S rRNA gene analysis.

DNA was extracted from bacterial isolates using freeze/thaw cycles to facilitate cell lysis. Amplification of the 16S rRNA gene was performed by PCR using the primers 616F (5’-AGAGTTTGATYMTGGCTCAG-3’; [35]) and 1510R (5’-GGCTACCTTGTTACGACTT-3’ [36],). The PCR reactions were carried out in a Biometra T-Gradient ThermoBlock thermocycler (Göttingen, Germany) with the following cycling parameters: 94ºC for 2 min,followed by 35 cycles of 94ºC for 20 seg, 55ºC for 20 seg and 72ºC for 2 min; and a final extension cycle at 72ºC for 10 min. The amplified products were evaluated by electrophoresis on 1% (w/v) agarose gel, stained with SYBR Safe DNA Gel Stain (Carlsbad, USA) and visualized under UV light. The PCR products were then purified and sequenced by STAB VIDA Sequencing Services (Caparica, Portugal). DNA sequences were edited in Bioedit v7.2.5 software (Technelysium, Tewantin, Australia) by visual inspection of chromatograms, trimming low-quality regions, removing primer sequences, and resolving ambiguous base calls. Phylogenetic identification was determined using the global alignment algorithm on the EzBioCloud database [37]. The generated 16S rRNA gene sequences were deposited in GenBank (https://www.ncbi.nlm.nih.gov/genbank/) under accession numbers PP902169-PP902183.

### Amplicon sequencing, taxonomic assignment and functional inference

Total genomic DNA was extracted from approximately 250 mg of each sample using the DNeasy PowerSoil Pro Kit (Qiagen) following the manufacturer's protocol. Lysis of the samples was performed using a FastPrep-24 homogenizer (MP Biomedicals). The quality and quantity of the extracted DNA were assessed using a Qubit 4.0 fluorometer (Thermo Scientific) and agarose gel electrophoresis. The V3-V4 hypervariable region of the prokaryotic 16S rRNA gene was amplified by PCR reactions using the universal primer pair 341F (5′- CCTACGGGNGGCWGCAG -3′) and 805R (5′- GACTACHVGGGTATCTAATCC -3′) with Illumina adapter overhang sequences [38]. Libraries were sequenced on an Illumina MiSeq platform and 250 bp paired-end reads were generated by Novogene Europe Sequencing Services.

Raw amplicon sequence data were processed in QIIME 2 version 2023.9 [39]. The imported paired-end reads were quality filtered, denoised, and merged using DADA2 plugin to generate an Amplicon Sequence Variant (ASV) feature table [40]. Taxonomic assignment of ASVs was performed using the SILVA reference database, version 138.2 (https://www.arb-silva.de,[41]). Alpha and beta diversity metrics were calculated in QIIME 2 to characterize within- and between-sample microbial diversity.

Functional Annotation of Prokaryotic Taxa (FAPROTAX; [42]) was used to infer putative functional profiles associated with the detected prokaryotic taxa, focusing on the main pathways involved in some of the major biogeochemical cycles. As FAPROTAX relies exclusively on taxonomic information from partial 16S rRNA gene sequences, the predicted functions should be considered hypothesis-generating and not evidence of actual metabolic activity.

The raw reads were deposited into the NCBI Sequence Read Archive (SRA) database under project ID PRJNA1167275.

### Statistical analysis

Beta-diversity patterns among lava tube samples were explored using Principal Coordinates Analysis (PCoA) based on a phylum-level Bray–Curtis dissimilarity matrix and a genus-level Bray–Curtis dissimilarity matrix calculated from the corresponding relative-abundance tables. Associations between environmental and mineralogical variables (temperature, relative humidity, CO₂ concentration, *δ*^13^C-CO₂, CH₄ concentration, water vapor condensates, and dominant mineral phases) were evaluated by fitting vectors onto the PCoA ordinations using envfit (vegan package), with significance assessed by permutation tests (999 permutations). Ordination biplots were produced in R using ggplot2 [112]. In addition, to assess the relationships between microbial diversity, mineralogical composition, and environmental conditions in the recently formed lava tubes, we performed a Pearson correlation analysis using the combined dataset of physicochemical variables (temperature, relative humidity, CO₂ concentration, *δ*^13^C-CO₂, CH₄ concentration, *δ*^13^C-CH₄), mineral abundances (thenardite, aphthitalite, augite, burkeite, trona, hanksite, and water vapor condensates), and alpha-diversity metrics (ASVs, Chao1, Shannon, Simpson). Pearson correlation coefficients (*r*) and associated significance values (two-tailed *p*-values) were calculated pairwise for all variables using the “scipy.stats.pearsonr” function in Python (v.3.11). Correlation matrices were generated separately for (i) *r* values, (ii) *p*-values, and (iii) a combined matrix reporting both metrics in the format *r* (*p*).

Network analysis was applied to examine bacterial and archaeal co-occurrence patterns, identify keystone species, and analyze the bacterial community structure in samples from lava tubes. Spearman's rank correlation coefficients were calculated using the average abundance of genera with relative abundances above 0.1%. Only correlations with a coefficient r > 0.6 and *p*< 0.01 were considered. In the reconstructed network, nodes represented genera, while edges indicated positive or negative correlations between them. Key topological properties, including network density, modularity, clustering coefficient, average degree, and average path length, were analyzed alongside betweenness centrality metrics. The results were visualized using Gephi 0.9.7 [43]. Associations within unidentified bacterial networks were modeled as symmetric, undirected connections.

To further evaluate the relative contribution of stochastic versus deterministic processes during early microbial colonization, we applied complementary community assembly approaches based on phylogenetic structure and abundance-occupancy patterns. Phylogenetic clustering within samples was assessed using the nearest taxon index (NTI), calculated as the standardized effect size of the mean nearest taxon distance (MNTD) relative to a permutation-based null model [44, 45]. Positive NTI values indicate phylogenetic clustering, whereas negative values indicate phylogenetic overdispersion, with |NTI|≥ 2 commonly interpreted as a strong deviation from random expectations. In addition, we fitted Sloan’s neutral community model to relate each taxon’s mean relative abundance in the metacommunity to its frequency of occurrence across samples, estimating the immigration parameter (m) and the expected occurrence frequency with confidence intervals [46]. Taxa were classified as falling within, above, or below the neutral expectation, and model performance was evaluated using the coefficient of determination (R^2^).

## Results

### Microenvironmental conditions in the newly formed lava tubes

The investigated lava tubes showed marked differences in morphology and microenvironmental conditions (Table 1). Overall, air temperature ranged from 18 °C to > 55 °C, and relative humidity was inversely related to temperature, reaching ~ 70% in cooler tubes and dropping to ~ 20–25% in the warmest sections (Table 1). Cave-air CO₂ concentrations were consistently higher than in the external atmosphere (520–720 vs. 440–463 ppm), and *δ*^13^C–CO₂ values spanned a broad range inside the tubes (− 14.5 to − 1.1‰) compared with outside air (− 11.8 to − 10.3‰; Table 1), indicating contrasting gas sources and ventilation regimes among tubes.

Among sites, *Sima Hornitos* and *Canal Hornito Bonito*, located in the northern sector of the lava field, exhibited the lowest air temperatures (18–21 °C) and highest relative humidity (65–72%). In contrast, *Tubo Rojo*, situated ~ 1 km downslope from the cone, remained thermally extreme, with air temperatures exceeding 55 °C, wall temperatures > 90 °C, and with the lowest relative humidity (~ 24%) and the highest *δ*^13^C–CO₂ measured in cave air (down to − 1.1‰; Table 1). Shatter Ring-1 displayed intermediate conditions during the 2023 campaign (26–28 °C air; 37–40 °C rock surface). Temperature monitoring further revealed pronounced temporal variability in *Sima Hornitos* and *Canal Hornito Bonito*, consistent with ventilation effects and residual geothermal heat (Figs. S2, S3).

### Mineralogy and structure of sulfate-rich minerals from newly formed lava tubes

**Fig. 3:**
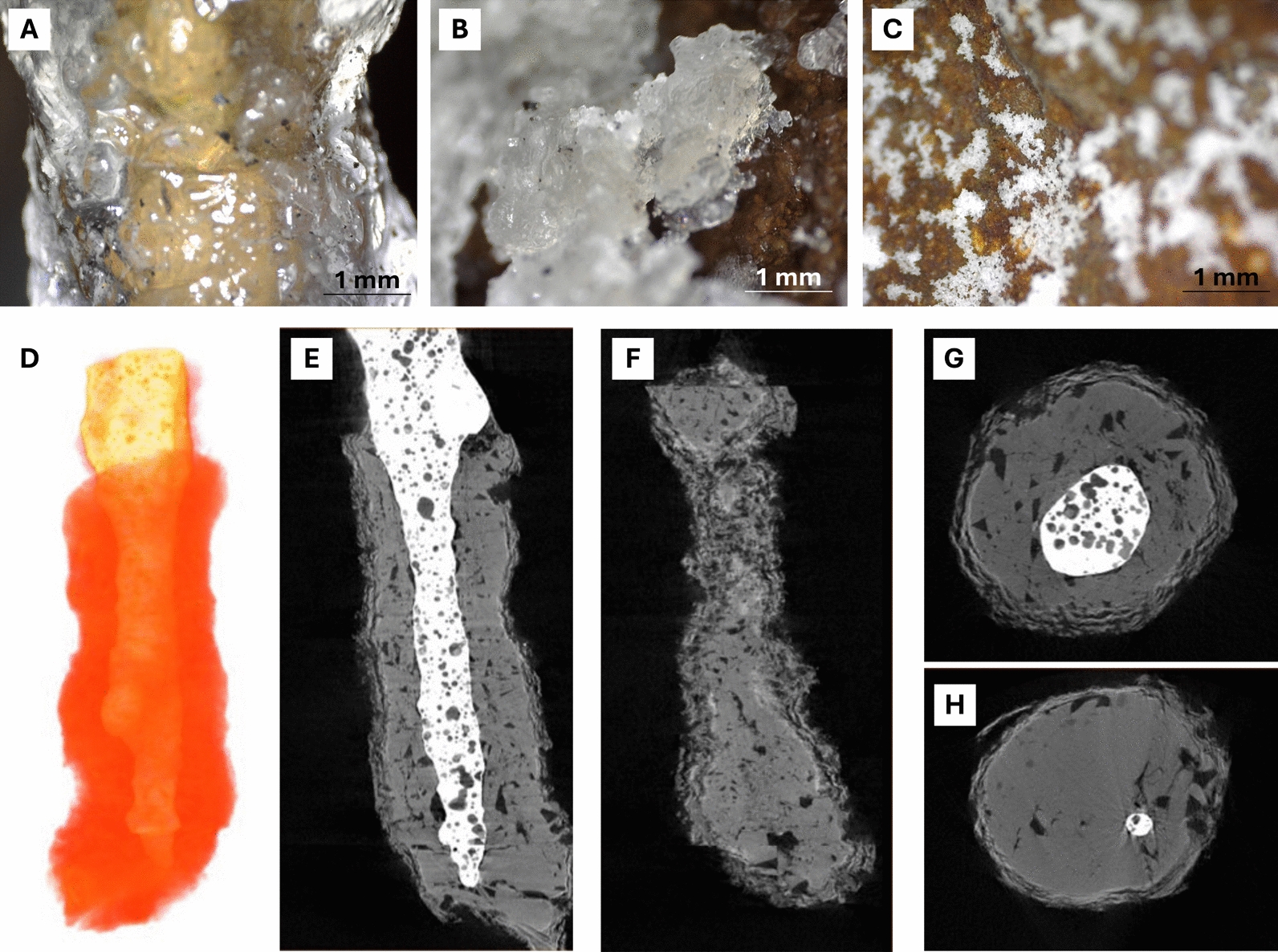
Field microscopy images of mineral deposits (**A–C**) and micro-CT reconstructions of a saline deposit from THB developed around a lava-based stalactite (**D–H**). **A** crystalline stalactites; **B** crusts; **C** powdery deposits coating lava surfaces; **D** 3D reconstruction with transparent layers (false color); **E** longitudinal section of the sample (white: lava; grey: salt deposit); **F** lateral section of the salt precipitate; **G, H** cross-sections of stalactite-type deposits

Salt precipitates occur on the walls and ceilings of the lava tubes as crystalline stalactites (Fig. 3A), crusts (Fig. 3B), and powdery deposits (Fig. 3C). Micro-CT imaging of a translucent stalactite from *Canal Hornito Bonito* Lava Tube (THB) showed that the most developed speleothems form around lava-based cores (Fig. 3D–H) and display alternating concentric layers differing in porosity and mineral phases (Fig. 3E–H). Bright areas in the micro-CT images correspond to dense lava-based material, whereas grey areas represent salt precipitates. The concentric outermost layering visible in the cross-sections (Fig. 3G, H) emphasizes the dynamic processes of mineral deposition.

The whitish speleothems abundantly observed in the newly formed lava tubes consist mainly of sodium sulfates and bicarbonates, predominantly thenardite (Na_2_SO_4_), trona (Na_2_CO_3_·NaHCO_3_·2H_2_O) and burkeite (Na_6_(CO_3_)(SO_4_)_2_), with minor aphthitalite ((K,Na)_3_ Na(SO_2_)_2_) and hanksite (Na_22_K(SO_4_)_9_(CO_3_)_2_Cl), as revealed by XRD (Table S2).

**Fig. 4:**
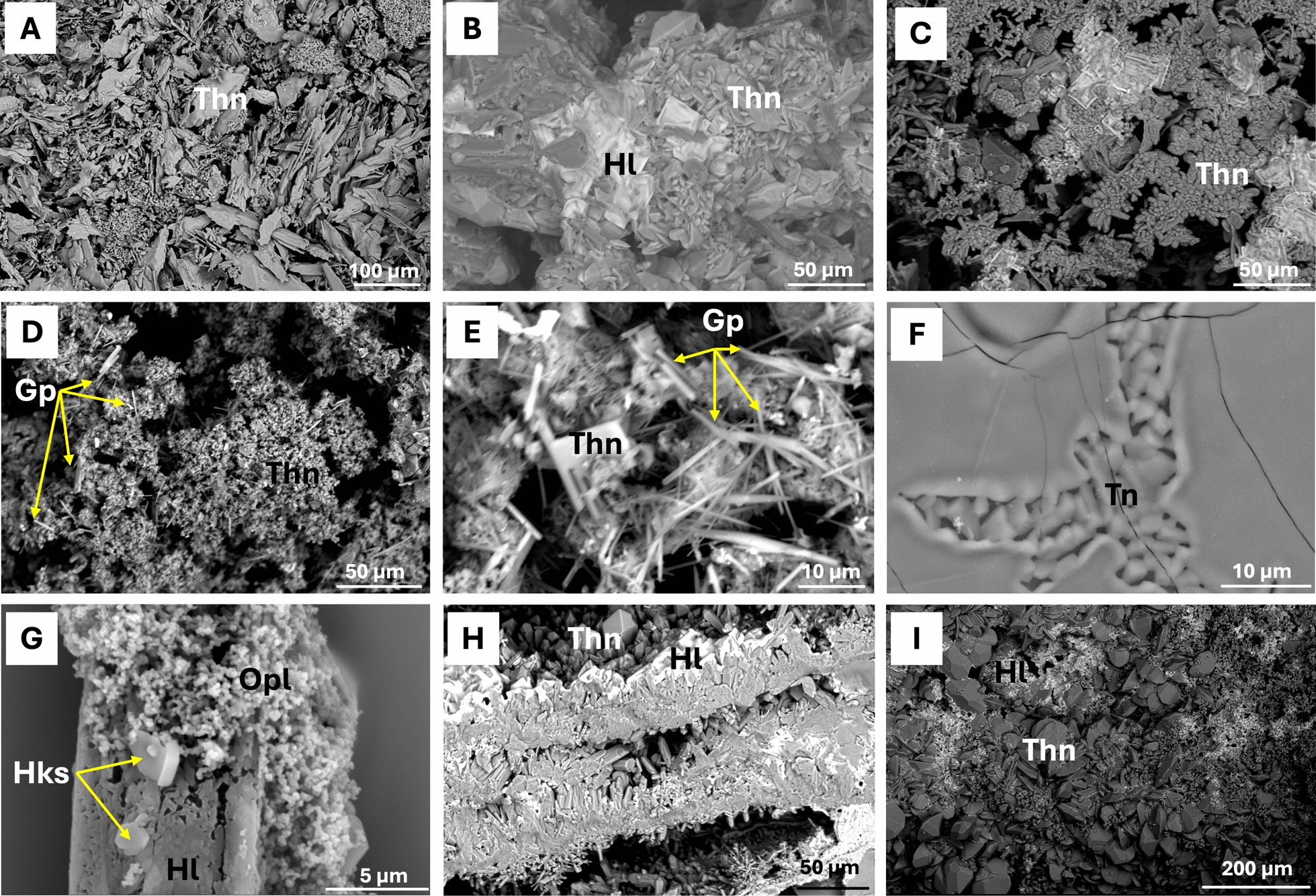
SEM photomicrographs of mineral deposits from the studied lava tubes, illustrating variations in texture and mineral composition: **A** Lamellar aggregates of thenardite (Thn) in *Hornito Bonito Channel* Lava Tube; **B** Halite (Hl) and thenardite in *Canal Hornito Bonito* Lava Tube; **C–E** Acicular gypsum (Gp) crystals embedded in thenardite aggregates from *Sima Hornitos*; **F** aggregate of trona (Tn) crystals from *Shatter Ring-1*, **G** Opal microspheres (Opl) associated with hanksite (Hks) and halite (Hl), from *Shatter Ring-1*; **H, I** Halite and sodium thenardite from *Tubo Rojo* Lava Tube

In *Sima Hornitos* and *Canal Hornito Bonito*, the translucent stalactites are dominated by dodecahedral and bipyramidal thenardite crystals (Fig. 4A–E), with aggregates of halite cement (Fig. 4B) and acicular gypsum identified by SEM–EDS (Fig. 4D, E). XRD analyses confirm sodium sulfate as the main phase (Table S2).

*Shatter Ring-1* speleothems exhibit a different mineralogy, consisting primarily of trona (~ 90%; Na_2_CO_3_·NaHCO_3_·2H_2_O) with minor hanksite (~ 10%; Na_22_K(SO_4_)_9_(CO_3_)_2_Cl) (Table S2). SEM–EDS shows trona microcrystals and subeuhedral hanksite associated with opal microspheres (< 1 µm) on altered halite surfaces (Fig. 4G).

In *Tubo Rojo* Lava Tube (sample TR0E), a layered deposit dominated by thenardite was observed, with halite filling cavities and interstitial spaces (Fig. 4H, I). Aphthitalite ((K,Na)_3_Na(SO_4_)_2_) and minor augite ((Ca,Na)(Mg,Fe,Al,Ti)(Si,Al)_2_O_6_) were also detected. Samples TR01 and TR02 were dominated by burkeite (Na_6_(CO_3_)(SO_4_)_2_) as revealed by XRD (Table S2).

### First detection of microbial features

**Fig. 5:**
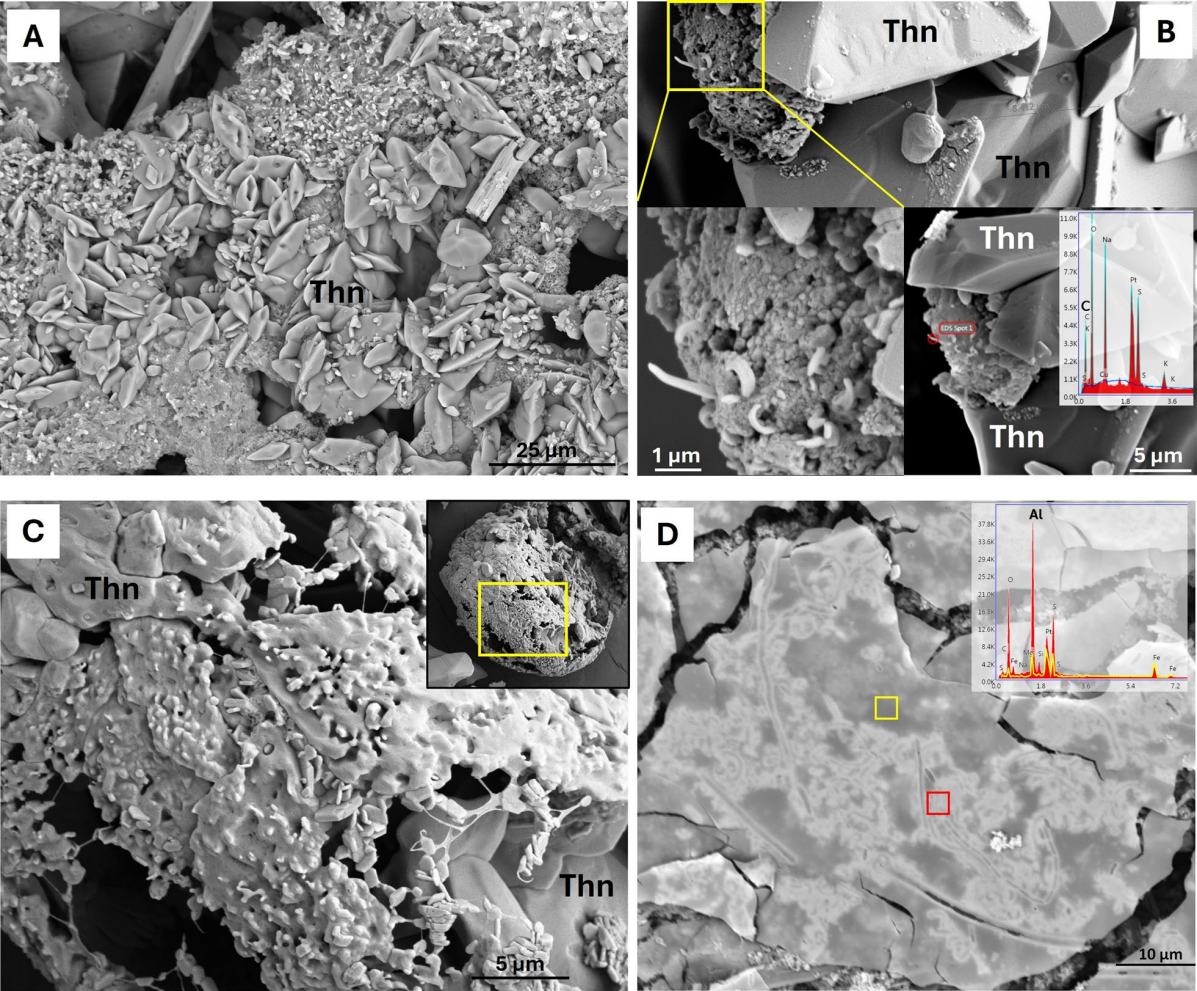
FESEM images of sample TSH1 of *Sima Hornitos*. **A** top left: subidiomorph crystals of thenardite (Na_2_SO_4_) with traces of Cu; **B** composite of three different magnifications of an organic cluster with microorganisms embedded in thenardite minerals, and EDS spectrum revealing C content, as well as S, O and Na, indicative of thenardite; **C** biofilm-like structure englobing large subidiomorph minerals 5–10 μm and smaller idiomorph crystals (1–3 μm); and **D** biofilm-like structure featuring an accumulation of Al (red EDS spectrum) in the bright biofilm-like areas compared to the plain dark areas (yellow EDS spectrum)

The whitish crusts, powder and stalactite samples were analysed by FESEM-EDS to get insights into the presence and morphology of microorganisms. Among all the samples, only those from *Sima Hornitos* (TSH1), composed of thenardite crystals (Fig. 5A), showed clear evidence of microorganisms and EPS (Fig. 5B–D). Spiral-shaped bacterial cells were observed embedded in a conglomerate of thenardite minerals (Fig. 5B). A glue-like biofilm matrix was observed in close association with mineral grains forming an organic-like spheric structure (Fig. 5C). Hexagonal crystals (1–3 μm) with tabular habit (idiomorph crystals) are cemented in the organic-like spheric structure, which also contains larger (up to 10 μm) subidiomorph minerals (Fig. 5C). In addition, a biofilm-like laminar crust of approximately 100 μm shows abundant rod-shaped cells and filamentous bacteria (Fig. 5D). This biofilm has an anomalous enrichment in Al and S detected in the bright microorganisms-shaped areas (red EDS spectrum in Fig. 5D).

### Microbial community composition

#### Isolated strains

A total of 13 isolates, identified as members of the *Bacillota* (60%) and *Actinomycetota* (40%) phyla (Table 2)*,* were obtained from samples collected in *Canal Hornito Bonito* Lava Tube (THB2), *Tubo Rojo* (TR01 and TR02), and *Shatter Ring-1* (TDS1-01). In *Shatter Ring-1* (TDS1-01), a more diverse culturable bacterial community was detected. The *Bacillota* phylum was present in both TDS1 and THB2 samples, while *Actinomycetota* was exclusively found in TR01, TR02, and THB2 samples.

**Table 2:** Taxonomic identification of the isolated bacterial strains

Lava tube	Strain ID (Accession number)	Nearest relative (Accession number)	Similarity (%)
*Canal Hornito Bonito*	THB2 s1	*Nocardioides cavernae* (KX815990)	99.38
	THB2 s5	*Priestia aryabhattai* (EF114313)	100.00
	THB2 s2	*Psychrobacillus psychrodurans* (jgi.1085849)	99.91
	THB2 s4.1	*Psychrobacillus vulpis* (MH910346)	98.90
*Tubo Rojo*	TR02 s1	*Arthrobacter tumbae* (AJ315069)	99.73
	TR01n s1	*Micrococcus luteus* (CP001628)	99.56
	TR01n s2	*Rathayibacter festucae* (CP028137)	99.82
*Shatter Ring-1*	TDS1-01 s6.2	*Alkalihalobacillus ligniniphilus* (ANNK01000138)	98.05
	TDS1-01 s5	*Filibacter tadaridae* (MK290395)	98.89
	TDS1-01t s1	*Paenibacillus lautus* (BIMF01000051)	99.91
	TDS1-01 s4.2	*Paenibacillus* sp. (KC978082)	95.18
	TDS1-01 s2	*Peribacillus butanolivorans* (LGYA01000001)	98.99
	TDS1-01 s3	*Peribacillus simplex* (BCVO01000086)	99.83

Four genera of the *Bacillota* were isolated from the TDS1-01, namely *Alkalihalobacillus, Filibacter, Paenibacillus* and *Peribacillus,* and two from THB2: *Priestia* and *Psychrobacillus.* In TDS1-01, *Alkalihalobacillus ligniniphilus*, a halotolerant bacterium previously found in marine environments [47, 48], and *Filibacter tadaridae*, originally isolated from bat guano in caves [49], were recovered. Additionally, *Paenibacillus lautus* (99.91%) and *Paenibacillus* sp. (95.18%) were identified, which are known for their ecological versatility and potential for bioremediation. *Peribacillus butanolivorans*, a species used industrially for butanol remediation [47], was also detected in this sample.

The THB2 sample yielded *Psychrobacillus psychrodurans* and *Psychrobacillus vulpis*, both psychrotolerant species [68, 107]. A strain of *Priestia aryabhattai* was also isolated, notable for its potential use in metal remediation [51, 52].

Four genera from the *Actinomycetota* phylum were identified: *Rathayibacter*, *Micrococcus*, *Arthrobacter*, and *Nocardioides*. *Rathayibacter festucae*, a plant pathogen that causes gummosis in grasses and cereals and is transmitted by nematodes of the genus *Anguinina* [53], was isolated from TR01, while *Micrococcus luteus*, a ubiquitous chemoorganotroph commonly found in lava tubes [95, 101], was identified in TR01. *Arthrobacter tumbae*, originally isolated from mural paintings from underground environments [56], was detected in TR02. *Nocardioides cavernae*, which thrives in nutrient-limited environments such as caves [57], was isolated from THB2.

Of particular interest was the isolation of *Paenibacillus* sp. (*Bacillota* phylum) from TDS1-01, which exhibited low similarity (95.18%) to 16S rRNA gene sequences in existing databases [58–60]. Given this low similarity, this strain is a candidate for further taxonomic studies to potentially classify it as a new bacterial species. Moreover, future whole-genome sequencing and comparative genomic analyses of these isolates will be essential to infer their metabolic potential and functional roles, and to link phenotypic observations with underlying genomic adaptations.

#### Prokaryotic richness and diversity

**Fig. 6:**
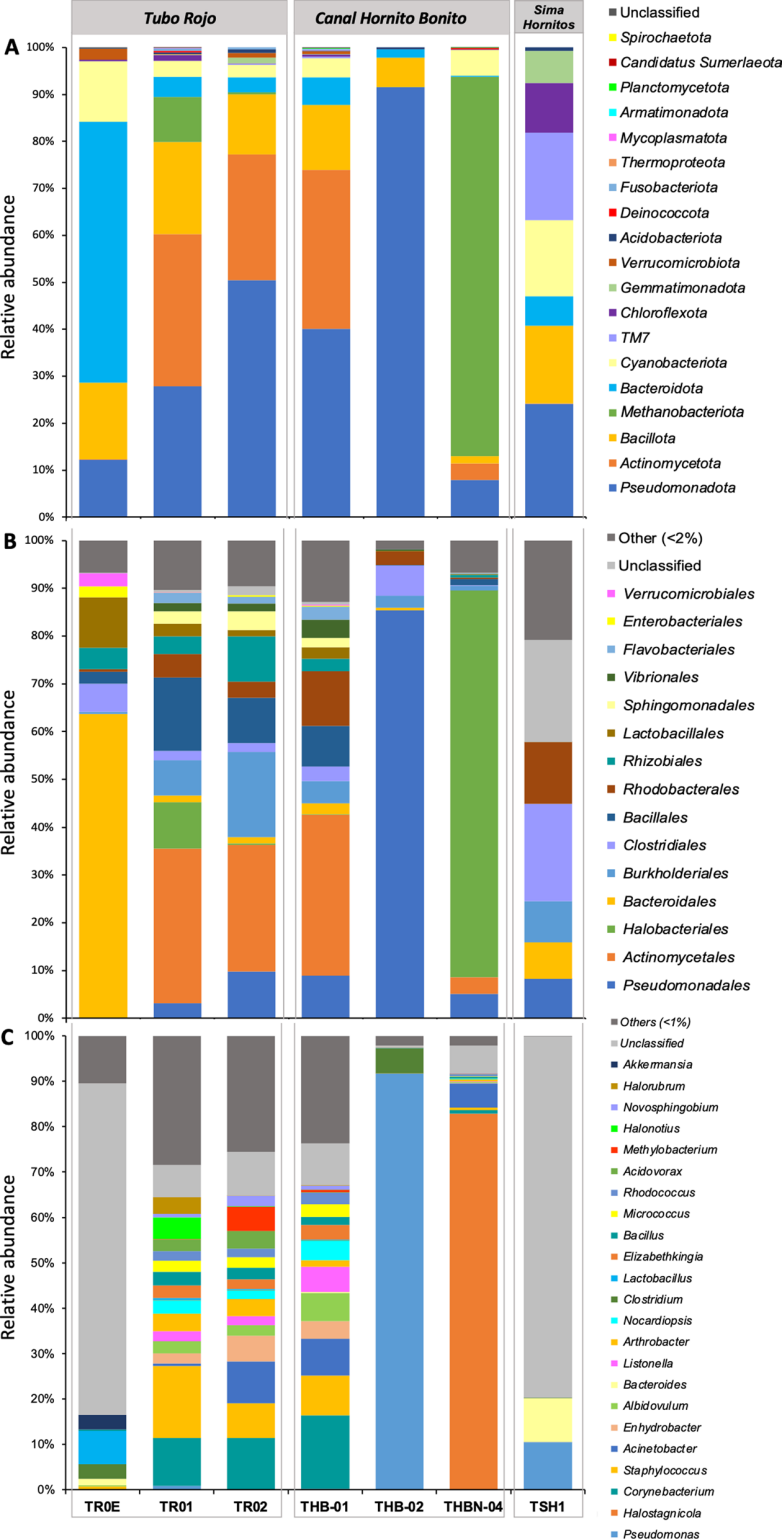
Prokaryotic community composition of the newly formed lava tubes. Barplots represent the relative abundances at the taxonomic levels: **A** Phylum; **B** Order, and **C** Genus

A total of 215,039 filtered DNA sequences were obtained from seven lava tube samples following Illumina MiSeq sequencing of PCR-amplified 16S rRNA genes (Table 3). The samples were collected from three distinct lava tubes. The number of amplicon sequence variants (ASVs) ranged from 14 in sample THB-02–465 in THB-01. TR0E was the most diverse sample, as indicated by the highest Shannon and Simpson index values. In contrast, samples THB-02, THBN-04, and TSH1 exhibited lower diversity indices and were dominated by specific bacterial groups, as revealed by the taxonomic composition analysis (Fig. 6).

**Table 3:** Alpha-diversity metrics of microbial communities across lava tube samples, including sequencing depth (filtered reads), observed and rarefied ASV richness, and diversity indices (Chao1, Shannon, and Simpson)

Lava tube	Samples	Filtered reads	ASVs	Rarefied ASVs	Chao1	Shannon	Simpson
*Tubo Rojo*	TR0E	18,411	167	162	167	6.44	0.98
	TR01	33,652	278	273	278	5.70	0.85
	TR02	42,560	216	184	216	6.12	0.93
*Canal Hornito Bonito*	THB-01	35,337	465	394	465	5.56	0.84
	THB-02	9250	14	14	14	1.20	0.30
	THBN-04	57,283	321	224	321	2.22	0.46
*Shatter Ring-1*	TSH1	18,546	20	19	20	3.16	0.87

#### Prokaryotic community composition and predictive functional profiling

The sequencing of the V3-V4 region of the 16S rRNA gene from the seven samples revealed the presence of eleven major phyla across *Tubo Rojo*, *Canal Hornito Bonito* and *Sima Hornito*, where *Pseudomonadota Actinomycetota*, *Methanobacteriota, Bacillota,* and *Bacteroidota* were the most dominant phyla (Fig. 6A). *Pseudomonadota* showed particularly high relative abundance in THB-02 (91.5%) and TSH1 (24.2%) samples. It is worth mentioning that TR01, TR02, and THB-01 also contain relatively high abundance (27.8 to 50.5%) of the *Pseudomonadota* phylum, with a majority of members belonging to the *Pseudomonadales*, *Burkholderiales, Rhodobacteriales* and *Rhizobiales* orders (Fig. 6B).

The *Actinomycetota* phylum was presented in similar proportions in TR01 (32.4%) and TR02 (26.7%) from *Tubo Rojo*, and in THB-01 (33.8%) from *Canal Hornito Bonito* Lava Tube, with the predominance of the *Corynebacterium* genus in all these samples (Fig. 6A, C). Interestingly, the THBN-04 sample was dominated by the archaeal phylum *Methanobacteriota*, comprising over 80.7% of the community (Fig. 6A), with the genus *Halostagnicola* representing the majority (82.9%). Members of this phylum were also found in TR01 but at significantly lower proportions (9.5%; Fig. 6A), mainly identified as belonging to the genera *Halonotius* (4.7%) and *Halorubrum* (3.7%) (Fig. 6C).

Additionally, the composition of TR01, TR02, and THB-01 comprised high abundances (12.8–19.7%) of members belonging to the *Bacillota* phylum, with the dominance of the *Staphylococcus* genus (Fig. 6C). This phylum was also found to be present in TR0E (16.2%) and TSH1 (16.6%) lava tube samples (Fig. 6A). In TR0E, the *Lactobacillales* and *Clostridiales* orders (Fig. 6B) were found, with the dominance of the *Lactobacillus* and *Clostridium* genera, while in TSH1 solely the *Clostridiales* order was detected (20.3%) (Fig. 6B–C).

Members of the phylum *Bacteroidota* were abundant in sample TR0E from *Tubo Rojo* (55.5% of the microbial community). In contrast, the other studied samples exhibited lower relative abundances, ranging from 6.2 to 0.3%. The phylum *Cyanobacteriota* was predominantly detected in samples TR0E and TSH1, with relative abundances of 12.9% and 16.2%, respectively. Several additional phyla were mainly identified in the TSH1 sample from *Sima Hornitos*, notably *TM7* (18.7%), *Chloroflexota* (10.6%), and *Gemmatimonadota* (6.8%).

**Fig. 7:**
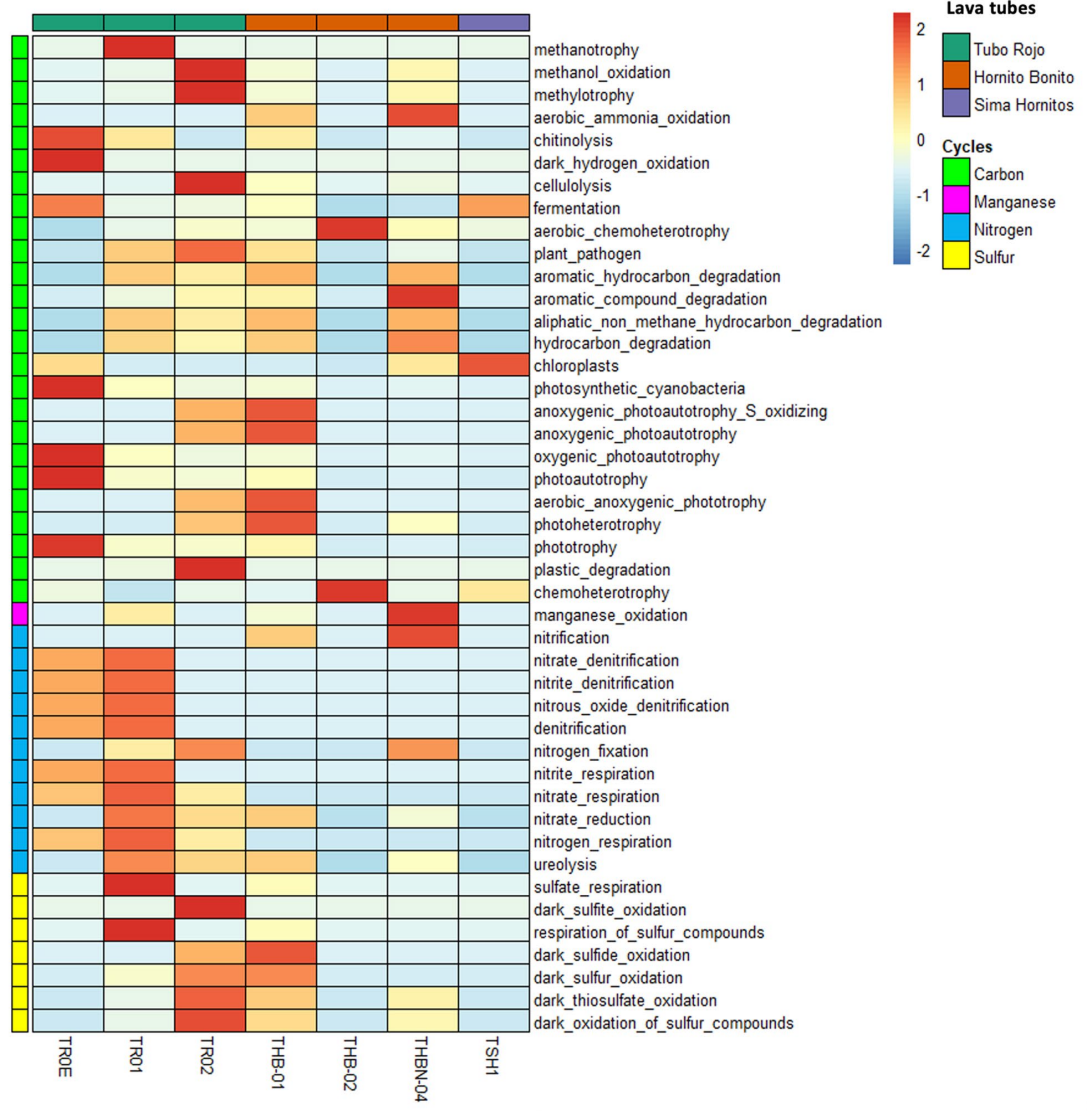
Heatmap showing the relative abundances of the putative microbial functional profiles inferred for each lava tube sample from the Tajogaite Volcano in La Palma, based on taxonomic assignments from 16S rRNA gene sequences using FAPROTAX. Values are displayed as z-score–standardized relative abundances (scale –2 to + 2), which highlight functional enrichment or depletion across samples

Regarding the carbon cycle inferred using FAPROTAX, chemoheterotrophy was the most widespread putative metabolic trait in THB-02 lava tube sample and also present in samples from TSH1, TR, and THB Lava Tube sites (Fig. 7). Methylotrophy, methanol oxidation and plastic degradation appeared mainly in TR02. Chloroplast-related activity was most abundant in TSH1 from *Sima Hornitos*, with lower representation in TR0E and THBN-04 (Fig. 7). In contrast, putative phototrophy-associated functions dominated in the *Tubo Rojo* and THB-01 samples. Fermentation was another commonly inferred function, particularly in TR0E and TSH1 samples.

For nitrogen-associated pathways, several putative functions were widely represented across *Tubo Rojo* samples, particularly in TR01. Nitrogen fixation appeared dominant in TR02 and THBN-04. In contrast, denitrification-related functions (nitrate-, nitrite- and nitrous oxide-denitrification) and nitrate/nitrite reduction and respiration were mainly associated with the *Tubo Rojo* samples, especially TR0E and TR01, while ureolysis showed higher representation in TR01 (Fig. 7). With respect to the sulfur cycle, functions related to sulfur compound respiration were inferred for TR01, whereas oxidative sulfur processes were more represented in TR02 and THB-01. Manganese oxidation was mainly associated with THBN-04.

Finally, predictive metabolic pathways associated with hydrocarbon and aromatic compound degradation were mainly represented in THB-01 and THBN-04 from *Canal Hornito Bonito* Lava Tube.

#### Multivariate community structure and assembly patterns

To identify associations between microbial community composition, environmental conditions, and mineralogical context, and to assess community assembly patterns in newly formed lava tubes, we combined Bray–Curtis-based ordination (PCoA) with environmental fitting (envfit), co-occurrence network analysis, and null-model–based assembly metrics.

**Fig. 8:**
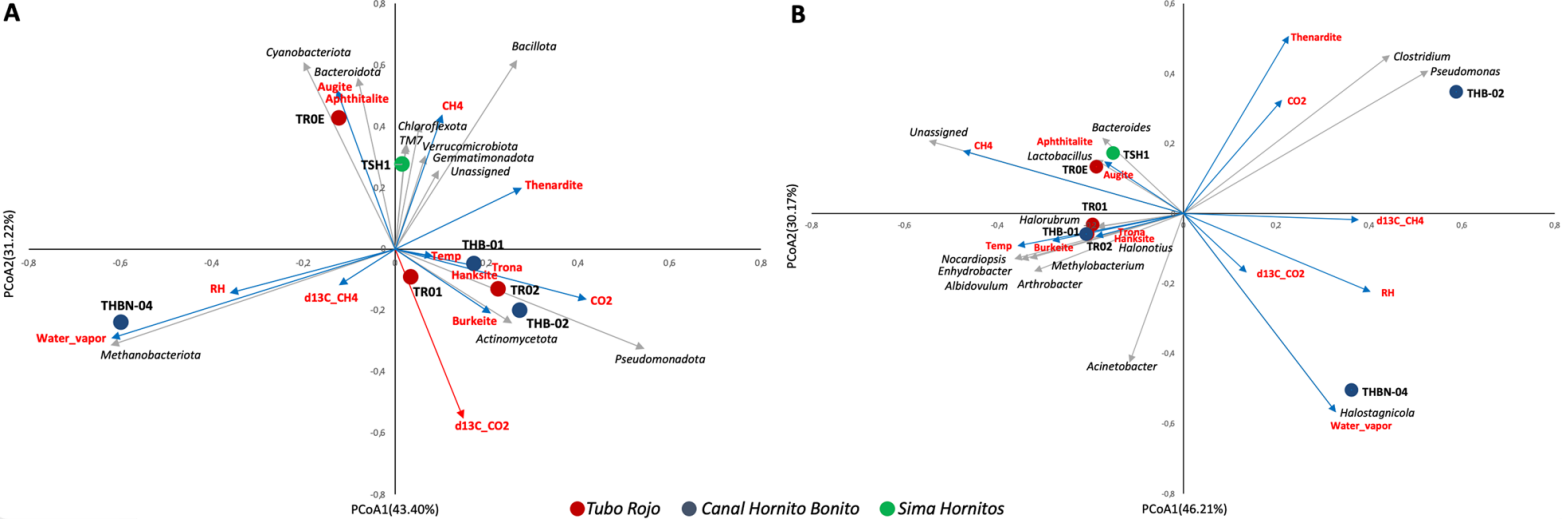
Faceted PCoA biplots showing sample ordination, taxon vectors, and fitted environmental and mineralogical variables at two taxonomic resolutions based on Bray–Curtis dissimilarities. **A** Phylum-level PCoA (PCoA1 = 43.40%, PCoA2 = 13.22% of variance explained). **B** Genus-level PCoA (PCoA1 = 46.21%, PCoA2 = 30.17% of variance explained). Points represent samples and grey arrows indicate taxon vectors. Environmental and mineralogical variables were fitted onto the ordination using *envfit*; red arrows indicate variables significantly associated with the ordination (*p* < 0.05), whereas blue arrows indicate non-significant variables (*p* ≥ 0.05)

At the phylum level, PCoA separated the samples along the first two axes (PCoA1 = 43.40%, PCoA2 = 31.22%; Fig. 8A), indicating marked differences in community composition across lava tube systems. *Canal Hornito Bonito* sample THBN-04 was clearly displaced towards negative PCoA1 and aligned with *Methanobacteriota* and the water vapor vector, suggesting a moisture-associated assemblage. In contrast, TR02 and THB-02 plotted towards positive PCoA1/negative PCoA2 and were associated with higher contributions of *Pseudomonadota* and *Actinomycetota*, in the direction of the CO_2_ vector and evaporitic mineral signatures such as Burkeite. TR0E separated from the main cluster towards positive PCoA2 and aligned with phyla such as *Bacteroidota* and *Cyanobacteriota* and Augite/Aphthitalite vectors, whereas TSH1 (*Sima Hornitos*) were associated with *Chloroflexota*, TM7, and other minor phyla (Fig. 8A). When overlaying environmental vectors, *δ*^13^C-CO_2_ was the only factor significantly associated with the phylum-level PCoA (red vector; *p* < 0.05) according to *envfit*, indicating that variation in *δ*^13^C–CO_2_ aligns with the main compositional gradient captured by the ordination (Fig. 8A). All other environmental variables (blue vectors) showed weaker, non-significant relationship with community structure (Fig. 8A).

At the genus level, PCoA explained a similar proportion of variation in microbial community composition (PCoA1 = 46.21%, PCoA2 = 30.17%; Fig. 8B) and revealed a clearer separation of several individual samples. THB-02 was strongly separated towards positive PCoA1/positive PCoA2 in the direction of genera such as *Pseudomonas* and *Clostridium*, coincident with vectors associated with CO_2_ and Thenardite. THBN-04 remained distinct, displaced towards positive PCoA1/negative PCoA2 and aligned with water vapor and *Halostagnicola*, reinforcing a haloarchaea-associated signature. By contrast, TR01, TR02, and THB-01 clustered closer to the origin, with contributions from multiple genera (e.g., *Nocardiopsis*, *Enhydrobacter*, *Albidovulum*, *Arthrobacter*) producing a more mixed central assemblage, while TR0E and TSH1 plotted towards negative PCoA1/positive PCoA2 near *Bacteroides* and *Lactobacillus* genera, and the Augite and Aphthitalite direction (Fig. 8B). Environmental fitting did not identify significant associations between the genus-level ordination and the measured environmental or mineralogical variables (*p* ≥ 0.05), indicating that the main compositional gradient in these nascent lava tubes was not structured by a single measured factor, but likely reflects combined influences and sample-specific community differences.

**Fig. 9:**
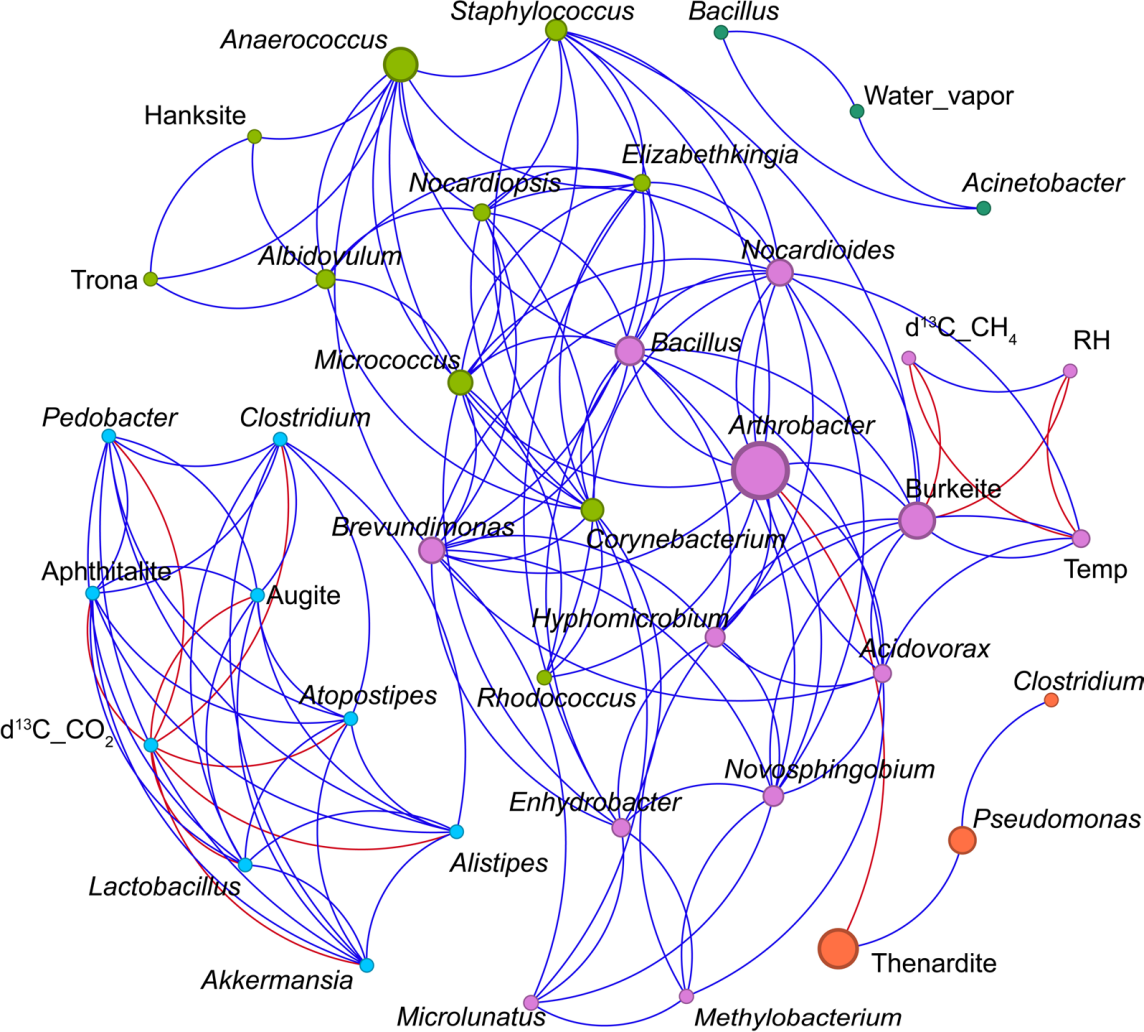
Network analysis showing the relationships among microbial genera (italicized names), minerals (thenardite, trona, hanksite, burkeite, aphthitalite and augite), environmental variables (Water vapor, RH, Temp) and isotope parameters (d^13^C_CH_4_ and d^13^C_CO_2_). Node size represents betweenness centrality, with larger nodes indicating core taxa or key connectors within the network. Blue lines represent positive correlations, and red lines represent negative correlations. The network is organized into five modules (colored by cluster) and reveals three distinct subnetworks

Network analysis based on betweenness centrality revealed key interactions between microbial taxa, minerals, and environmental variables (Fig. 9; Table S3). The size of each node in the network reflects its betweenness centrality, indicating its role in connecting different elements within the system. *Arthrobacter* emerged as the most central and highly connected genus, suggesting a key ecological role across various microhabitats in the lava tubes. Other genera with high centrality included *Anaerococcus Bacillus*, *Pseudomonas*, *Nocardioides*, and the minerals such as thenardite and burkeite, all of which function as important connectors between microbial groups, mineral assemblages, and environmental gradients. In the smaller subnetworks, the nodes appear to be well connected, with none serving as bridges to enhance their connectivity.

Environmental variables such as temperature and relative humidity, and stable isotope values (*δ*^13^C-CO_2_ and *δ*^13^C-CH_4_) were negatively associated with specific microbial genera (Fig. 9), highlighting that these factors did not appear to strongly influence overall network connectivity, as they showed predominantly negative correlations with most microbial taxa. Notably, Shannon and Simpson indices were also associated negatively with *δ*^13^C-CH_4_ (Table S3). Microbial alpha-diversity metrics exhibited positive correlations with trona- and hanksite-rich samples, but negative relationships with thenardite and increasing humidity (Table S3). The nodes in the network seem well interconnected, as indicated by the high average degree (7.05) and density (0.181) values, despite the network's compartmentalization into three subnetworks (Fig. 9). The density values correspond to moderate modularity, with five distinct modules. The high average path length (2.278) suggests that while the community is generally well-connected, nodes require multiple intermediate steps to connect due to a stretched network structure. This is particularly evident in the largest subnetwork. The high average clustering coefficient (0.725) indicates that nodes form densely interconnected clusters, suggesting co-occurrence within specific subsets of the network. These cohesive subgroups may enhance resilience by providing alternative interaction pathways within local groups. In addition, such clustering often reflects cooperative interactions, such as mutualism or synergistic relationships within localized taxa. This pattern aligns with the strong positive correlations observed between taxa (90.78%, Fig. 9).

Since these networks consist of symmetric ties and undirected associations, and lack definitive patterns indicating clear synergistic or antagonistic relationships, making assumptions about bacterial and archaeal interactions may result in inaccuracies. Nevertheless, betweenness centrality highlights the significance of certain taxa, such as *Arthrobacter* and *Anaerococcus*, which act as bridges enhancing connectivity and, consequently, the resilience of the largest bacterial subnetwork. In contrast, such bridging roles are unnecessary in the smaller subnetworks, where nodes appear to form cohesive groups.

**Fig. 10:**
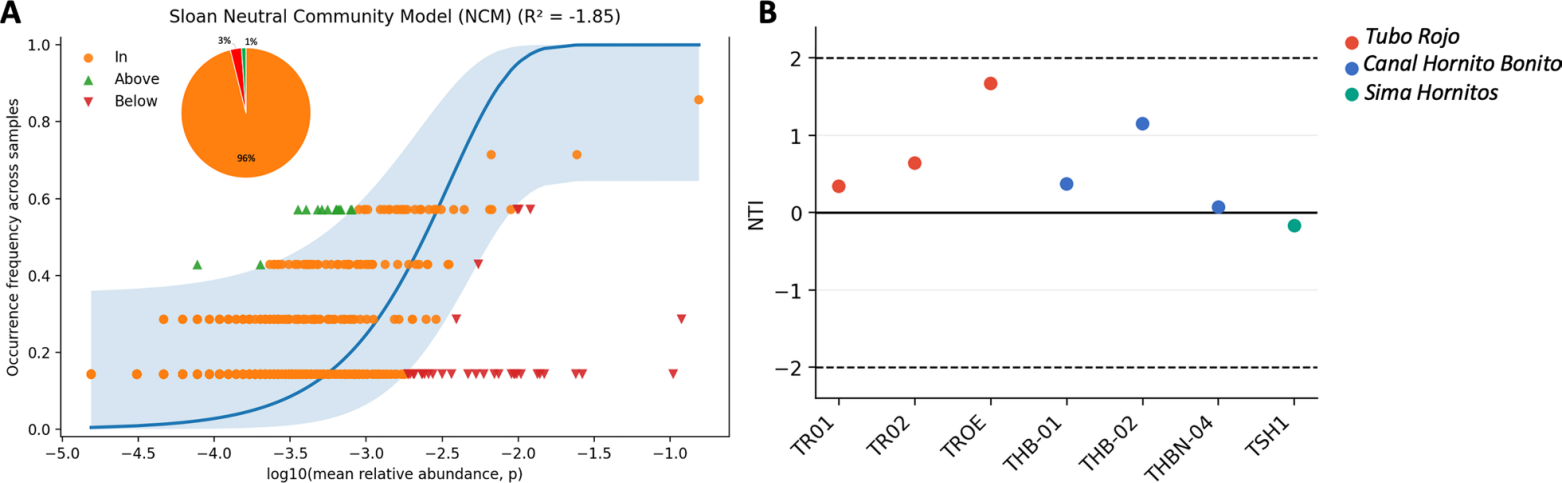
Community assembly signals in Tajogaite lava tubes (La Palma) supporting mixed stochastic–deterministic colonization. **A** Sloan neutral community model showing the relationship between each taxon’s mean relative abundance (log10 scale) and its occurrence frequency across samples. The solid line represents the neutral expectation and the shaded area the model confidence interval; taxa are classified as within (“In”), above, or below neutral predictions. The inset pie chart summarizes the proportion of taxa in each category (In, Above, Below). **B** Nearest Taxon Index (NTI) values per sample, colored by lava tube (*Tubo Rojo*, *Canal Hornito Bonito*, and *Sima Hornitos*). Horizontal dashed lines mark the commonly used threshold for strong deviation from null expectations (|NTI|≥ 2), while the solid line indicates NTI = 0

To explore whether early microbial colonization in the Tajogaite lava tubes was dominated by deterministic filtering or by stochastic processes, we examined within-sample phylogenetic structure (NTI) and fitted Sloan’s neutral community model (NCM) to the abundance–occupancy relationship. NTI values were low across all sites, ranging from − 0.17 to 1.67 (mean ≈ 0.58), and none of the samples exceeded |NTI|= 2, indicating no strong deviation from random phylogenetic structure within samples. Consistent with this phylogenetic structuring, the NCM plot shows that the vast majority of taxa fall within the neutral prediction envelope (“In”; Fig. 10A, inset pie chart), which supports a substantial contribution of stochastic processes compatible with neutral dynamics (e.g., ecological drift and probabilistic dispersal) to early community assembly across tube microhabitats. Importantly, this evidence should be interpreted as supportive rather than definitive, because neutral-like abundance–occupancy patterns can coexist with (or partially mask) deterministic effects. In this context, the smaller fractions of taxa that occur consistently above or below the envelope point to localized non-neutral departures, where “Above” taxa may reflect enhanced persistence/selection or higher effective dispersal, whereas “Below” taxa may be consistent with dispersal limitation and/or environmental filtering restricting establishment. Overall, the combined NTI and NCM results indicate predominantly stochastic assembly with a limited subset of taxa showing deterministic deviations among microhabitats.

## Discussion

### Evidence for bacterial and deep-seated CO_2_ contributions to the atmospheres of the Tajogaite lava tubes

Air temperatures and humidity varied strongly among the Tajogaite lava tubes, with cooler and more humid conditions near the cone (*Sima Hornitos* and *Canal Hornito Bonito*) and very warm, dry air in *Tubo Rojo* (Table 1). This elevated temperature about 1 km downslope from the cone, likely contributes to the markedly low humidity (~ 20%) and limited water availability observed in *Tubo Rojo*.

**Fig. 11:**
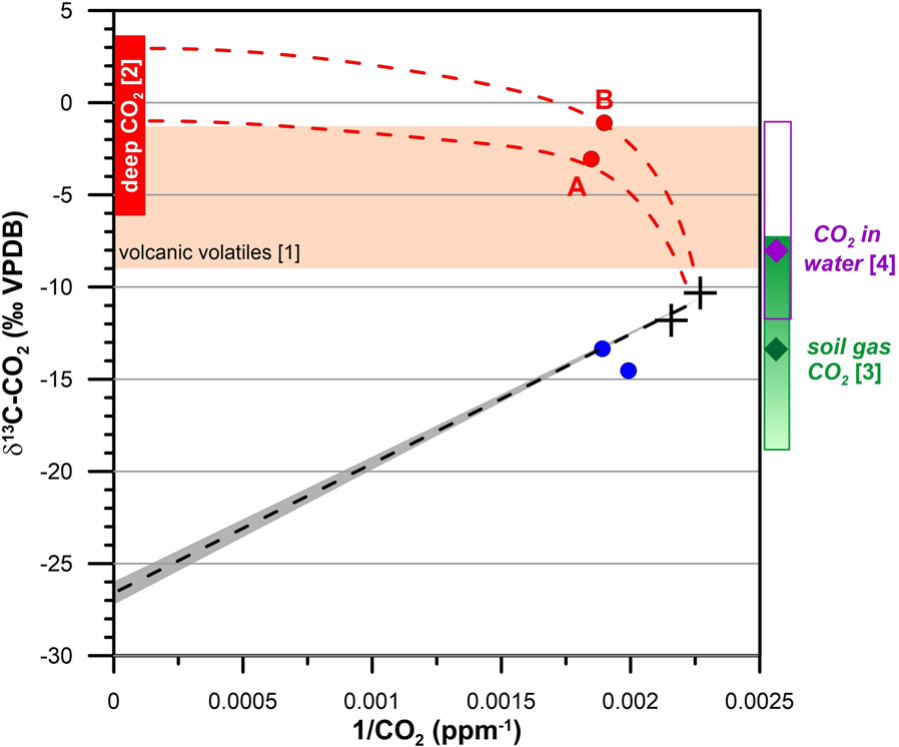
Interpretative Keeling plot with the preliminary CO_2_ air data set from the newly formed lava tubes after the 2021 eruption of the Tajogaite Volcano. Air samples: deep-seated CO_2_ (red circles, dashed red line represents the addition of deep-seated CO_2_), biogenic CO_2_ (blue circles) and CO_2_ of the local atmosphere (black crosses). The theoretical mixing of atmospheric and biogenic (soil-derived) CO_2_ is fitted with a linear keeling function (dashed black line and grey shaded area for uncertainties), considering the ^13^C-depleted end-member the *δ*^13^C values reported for the acid soils above older lava tubes on La Palma (–26.6 ± 0.4‰; [12]). Some reference ranges of *δ*^13^C-CO_2_ are considered for data discussion (see text): [1] typical range of *δ*^13^C-CO_2_ for arc-related volcanic volatiles [65] (orange shaded area), [2] theoretical *δ*^13^C-CO_2_ of deep CO_2_ based on gas emission of previous volcanic eruptions in La Palma Island [67] and from the 2021 Tajogaite eruption [63], [3]range of carbon isotopic composition in soil registered gases at Tajogaite Volcano, before, during and after the eruptive period [96] and [4] range of carbon isotopic composition of groundwater during the seismic activity and the last at the Tajogaite volcanic system [94]

CO_2_ concentrations inside the lava tubes were consistently higher than those of the outside atmosphere, indicating additional sources beyond simple atmospheric mixing. Keeling plot analysis (Fig. 11), which evaluates isotopic signatures by plotting the inverse of CO₂ concentration against its *δ*^13^C value [61, 62], revealed two distinct patterns. The first group, including samples from *Canal Hornito Bonito* and the midsection of *Tubo Rojo*, displayed *δ*^13^C-CO_2_ values within the range reported for Tajogaite soil-gas *δ*^13^C-CO_2_ before and during the eruption (–17.9 to –7.3‰, mean –13.4‰; [96]. Because these newly formed lava tubes lack organic surface layers or overlying soils, this depleted *δ*^13^C-CO_2_ signature is unlikely to result from the decomposition of surface organic matter and the subsequently gas diffusion to the lava tube environment. Several processes could account for the observed values. One possibility is isotopic CO_2_ fractionation occurring inside the lava tubes, potentially linked to early-stage microbial activity by pioneering microbial communities. Another plausible contribution is CO_2_ produced by thermal alteration of plant debris (and, locally, remobilized residual soil material) entrained and buried during lava emplacement, which can generate isotopically light CO_2_. Although additional analyses are required to distinguish among these potential sources, our data suggest that combined field-based gas surveys and stable-isotope approaches may help identify early microbial CO_2_-processing mechanisms in newly formed lava tubes.

The second group of samples, collected from *Sima Hornitos* and the entrance zone of *Tubo Rojo* (sites A and B in Fig. 11), showed markedly enriched *δ*^13^C-CO_2_ values, consistent with a deep endogenous volcanic contribution. Similar *δ*^13^C-CO_2_ signatures have been reported for CO_2_ in fluid inclusions hosted in olivine and clinopyroxene from the 2021 Tajogaite lavas [63], and in groundwater samples from the Peña Horeb gallery during the 2021 Tajogaite eruption [94]. These values match those of typical arc-related volcanic volatiles [65]. Comparable values have also been documented in soil gases from hydrothermal systems in other volcanic regions [66], further supporting a magmatic origin for the CO_2_ in these specific sites.

Based on these comparisons and the relatively low CO_2_ concentrations found in these tubes, we propose that the observed geogenic CO_2_ represents a residual magmatic signal that has undergone substantial mixing with atmospheric air. The distinctive *δ*^13^C-CO_2_ signature reflects continued volatile release from deep magmatic sources, preferentially venting through fracture networks and accumulating in the lava tube atmospheres. The inferred isotope composition of this deep-seated CO_2_ is consistent with previous measurements from older lava tubes on La Palma, where similar values were attributed to diffuse degassing and isotopic fractionation of magmatic CO_2_ [67]. Consistently, *envfit* analyses fitted onto PCA ordinations identified *δ*^13^C-CO_2_ as the only variable significantly associated with community turnover at the phylum level (Fig. 8A; permutation test, *p* < 0.05). This independent ecological signal reinforces the Keeling-plot inference, suggesting that CO_2_ source identity and/or in-tube CO_2_ processing covaries with broad-scale microbial community structure in the Tajogaite lava tubes, whereas absolute CO_2_ concentration and the remaining microclimatic/mineralogical variables were not significantly associated with the phylum-level ordination. Given the limited sample size, this relationship is interpreted as correlational and should be validated with expanded spatial and seasonal sampling.

### Substrate for microbial community colonization in the Tajogaite lava tubes

The surfaces within the Tajogaite lava tubes consist mainly of fresh basaltic glass, produced by rapid lava cooling particularly evident in *Tubo Rojo*, and secondary sulfate- and carbonate-rich deposits formed shortly after the eruption.

XRD analyses confirmed data that the substrates within the sampled lava tubes are dominated by highly soluble Na-rich evaporite minerals (Table S2), such as thenardite (Na_2_SO_4_), trona (Na_2_CO_3_·NaHCO_3_·2H_2_O), burkeite (Na_6_(CO_3_)(SO_4_)_2_), aphthitalite ((K,Na)_3_Na(SO_4_)_2_) and the mixed chloride–carbonate–sulfate mineral hanksite (Na_22_K(SO_4_)_9_(CO_3_)_2_Cl), which have been reported in other volcanic caves [69].

SEM–EDS analyses indicated that the mineral assemblages incorporate components derived from the host basalt consistent with the moderately Na-rich composition of the Tajogaite basalts (Na_2_O ~ 3–4 wt% [110]), as well as contributions from volcanic gases, suggesting abiotic formation followed by weathering through condensation rather than microbially induced processes [20].

Water availability in the tubes may also influence speleothem formation. Gravitational speleothems indicate dripping water, likely from rainfall infiltration, although the semi-arid conditions and low annual precipitation since the eruption (272 mm) suggest that condensation is probably the dominant water source, especially in the cooler tubes, either from volcanic degassing or from external humid air masses. The mineralogical and textural features observed in micro-CT and SEM imaging of the speleothems indicate that secondary minerals formed by a two-stage process: an early phase dominated by sublimation and deposition of hot volcanic gases during the cooling of the lava tubes, followed by later aqueous overprinting driven by condensation. This is consistent with the dense cemented inner areas of the stalactites (Fig. 3E–H) and the outer concentric layered precipitates that reflect aqueous deposition (outer layers, Fig. 3E–H).

Interestingly, Na-rich sulfate, carbonate, and mixed-salt minerals, such as thenardite, trona, burkeite, and hanksite, are highly soluble and, in some cases, contain structural water, allowing them to deliquesce or release hydration water under appropriate microclimatic conditions [55, 70]. The resulting brines are strongly alkaline and characterized by very high ionic strength (e.g., trona: ~ 420 g/L; thenardite: ~ 470 g/L at 20 °C), generating osmotic conditions comparable to saline environments [71, 72]. Such physicochemical conditions provide a compelling explanation for the prevalence of halophilic microorganisms observed in our microbial profiles, such as the archaeal genera *Halostagnicola*, *Halonotius* and *Halorubrum*, which are commonly associated with hypersaline environments [73]. Moreover, the potential release of liquid water through mineral hydration or deliquescence may offer transient but ecologically meaningful moisture sources capable of sustaining microorganisms adapted to osmotic stress, a mechanism previously proposed for other arid ecosystems [74, 75]. The abundance of these Na-rich mineral phases thus indicates that salinity-driven environmental filtering is likely a key factor structuring pioneer microbial communities in the newly formed lava tubes.

FESEM-EDS analyses revealed microbial-like filaments, rods and extracellular polymeric substances (EPS), consistent with early-stage biofilm formation. EPS are known to facilitate survival under desiccation and oligotrophic conditions and can influence mineral nucleation by concentrating ions and modifying local geochemistry [20, 76–78]. This aligns with the localized enrichments of aluminium, sulfur and trace copper (Cu), suggesting interactions between microorganisms and mineral substrates. EPS can enhance sorption of negatively and positively charged ions and create microenvironments that promote localized dissolution or reprecipitation, potentially concentrating mineral-derived Al and S from the basaltic host rock as well as from the Na-rich secondary mineral deposits at the biofilm-mineral interface [78, 79]. Cu is a critical cofactor for many microbial enzymes, including multicopper oxidases used to mitigate oxidative stress [99]. Copper minerals have been observed in other studies of Hawaiian and New Mexican lava tubes [10, 81]. Kopacz et al. [82] showed that localized Cu enrichments can occur through the infiltration of Cu-bearing groundwater derived from volcanic ash or hyaloclastite formations, leading to the formation of blue Cu-rich secondary minerals such as chrysocolla in Icelandic lava tubes. These observations suggest that biofilm-forming pioneer communities may already be contributing to early elemental cycling within the lava tubes.

### Evidence of early ecosystem development in nascent lava tubes

The microbial diversity observed in the newly formed lava tubes of La Palma indicates the early establishment of diverse and compositionally heterogeneous communities. The most abundant phyla were *Pseudomonadota*, *Actinomycetota*, and *Bacillota*, in line with previous findings in volcanic systems. For example, [4] reported similar phyla in Icelandic lava flows adapted to both cold conditions and geothermal activity, while Gutierrez-Patricio et al. [101] described comparable assemblages in lava tubes of Tenerife. Similarly, Azorean lava tubes host communities dominated by *Pseudomonadota*, *Bacillota*, and *Bacteroidota*, with additional representation of *Acidobacteriota*, *Nitrospirota*, and *Chloroflexota* [10]. These microorganisms are frequently reported in oligotrophic environments and are known to include taxa with broad metabolic versatility [83].

Natural light-exposed samples (TR0E and TSH1) showed high relative abundances of *Cyanobacteriota*, consistent with their frequent role as pioneer colonizers in cave entrances where photosynthesis is possible [4, 84]. *Bacteroidota*, particularly abundant in TR0E, include lineages often involved in the degradation of complex organic matter and in early nutrient cycling. The presence of diverse *Alphaproteobacteria* and *Betaproteobacteria* (within *Pseudomonadota*) further reflects the broad ecological range of these early microbial communities, including taxa previously reported to participate in nitrogen cycling, such as *Novosphingobium* (detected in TR02; Smit et al., [111]). *Gammaproteobacteria*, identified across several samples, include genera associated with organic matter degradation and tolerance to saline substrates (e.g., *Listonella*).

The phylum *Bacillota* was predominantly found in samples TR0E, TR01 and TSH1, mainly represented by *Methylobacterium*, *Bacillus*, and *Clostridium* genera, which include species reported from environments subject to temperature fluctuations, desiccation, or low nutrient availability [85, 86].

Previous studies by Riquelme et al. [11] and Herrera et al. [87] have shown that lava tubes host diverse microbial communities capable of colonizing volcanic glass and rock substrates. The presence of *Cyanobacteriota* and *Actinomycetota* in our samples aligns with their frequent occurrence in other basaltic cave systems [84]. In Hawai'ian lava tubes, *Actinomycetota*, *Pseudomonadota*, and *Acidobacteriota* are abundant and include taxa known to interact with volcanic minerals [10, 13]. These comparisons with other volcanic regions suggest that the microbial communities in the Tajogaite lava tubes share structural similarities with other recently formed basalt-hosted systems, while also displaying distinct features such as the unusually high abundance of Archaea, especially in samples THBN-04 (80.7%) and TR01 (9.5%).

While bacteria typically dominate mature lava tubes in Iceland, Hawai'i and the Azores [10, 13, 82, 88], archaeal-enriched communities have been reported in volcanic settings characterized by hypersaline crusts, hydrated sulfate minerals or severe oligotrophic conditions similar to those found in our study [73, 89, 90]. The dominant archaeal phylum *Methanobacteriota*, represented mainly by *Halostagnicola*, *Halonotius*, and *Halorubrum* and typically associated with hypersaline and desiccation-prone environments [73, 91, 92], is consistent with the Na-rich sulfate/carbonate mineralogical composition observed here. These minerals (e.g., thenardite, trona, burkeite, hanksite) create saline, hygroscopic and often alkaline microhabitats that can form thin brines through deliquescence [71]. These conditions, combined with the absence of surface soil-derived organic matter and early successional stages, may favor halophilic archaeal groups during the earliest phases of colonization. The pronounced abundance of *Methanobacteriota* detected here despite the use of universal 16S rRNA gene primers suggests that Archaea represent a major component of the pioneer microbiome in these newly formed lava tubes. Hathaway et al. [93] has demonstrated that archaeal-specific primers can significantly enhance the detection and resolution of archaeal communities in volcanic cave environments. Future studies combining universal and archaeal-targeted amplicon sequencing, or whole-genome approaches, will be essential to fully resolve archaeal diversity, population structure, and functional potential during early lava tube colonization.

Predicted functional profiles inferred from FAPROTAX (Fig. 7) suggest early ecosystem functions such as photosynthesis near cave entrances, nitrogen fixation, and organic matter degradation. However, these predictions must be interpreted with caution as FAPROTAX assigns functions based solely on taxonomic affiliation, and not on measured metabolic activity. In particular, confirming nitrogen fixation would require targeted detection of nitrogenase gene [50] and/or metagenomics and genome-resolved approaches. The presence of *Cyanobacteriota* as primary producers in light-exposed lava tube sections underscores their importance in early ecosystem development. Putative chemoheterotrophy, particularly in sample THB-01, is consistent with the detection of *Pseudomonas*, *Nocardioides*, and *Bacillus*, likely reflecting the reliance of early microbial assemblages on limited organic inputs in lava tubes [10]. The bacterial strains isolated from samples THB2, TR01, TR02, and TDS1-01, assigned to *Actinomycetota* and *Bacillota*, as well as the isolation of *Nocardioides cavernae* from THB2, a species known to occur in oligotrophic cave soils, reinforces the idea that early colonizers in these nascent lava tubes are capable of persisting under low-nutrient conditions. Predictive functions related to sulfur cycling in TR01, TR02 and THB-01 likely reflect the availability of sulfur-bearing minerals and organic matter associated with the basaltic substrate, which can support both sulfur oxidizing and reducing microorganisms [88].

### Animal-derived organic debris as potential drivers of microbial colonization

Our findings indicate that early microbial colonization in the Tajogaite lava tubes may also be influenced by animal-derived organic, particularly seabirds and small terrestrial fauna. The detection of genera commonly associated with animal microbiota, such as *Staphylococcus*, *Sphingomonas* and *Pseudomonas* (Fig. 6C), together with the isolation of *Psychrobacillus vulpis* and *Filibacter tadaridae* (Table 2), suggest animal-associated sources within the cave environments. The detection of *Rathayibacter festucae* (*Actinomycetota* phylum*)*, a nematode-associated plant pathogen, further suggests that invertebrates may contribute to microbial dispersal. These bacterial taxa may benefit from organic matter introduced by birds and rodents observed near cave entrances [104]. Field observations of pigeons and seabird guano deposits, feathers, and nesting materials in Tajogaite lava tube entrances reinforce this explanation (Fig. S1), although guano chemistry or animal DNA-based analyses were not performed in this study.

Global studies have shown that seabird colonies act as important biogeochemical hotspots, transferring large amounts of nitrogen and phosphorus from marine to terrestrial ecosystems [64]. The high concentrations of nitrogen in seabird fecal material (1–25% total N) may introduce bioavailable nutrient sources into the nutrient-poor conditions typical of newly formed volcanic environments, potentially contributing to the initial seeding and enrichment of microbial communities, and accelerating early ecosystem development. Beyond nutrient inputs, early colonization likely integrates multiple dispersal sources, including airborne microbes and dust, wind-blown debris and spores, and animal-mediated transport (e.g., seabirds, insects, cave-visiting fauna), which can introduce both microorganisms and organic substrates. In addition, episodic rainfall and runoff may further act as vectors for microbial dispersal and redistribution across newly formed lava tube surfaces. In Icelandic lava flows, Hadland et al. [23] showed that, after the first winter, rainwater became the predominant source contributing to lava microbial assemblages, underscoring precipitation as a key driver of community inputs over time.

### Stochastic seeding followed by deterministic filtering

Principal coordinates analyses with environmental fitting support a dual assembly scenario in these nascent lava tubes. At the phylum level *envfit* identified *δ*^13^C-CO_2_ as the only variable significantly associated with the PCoA structure (Fig. 8A), consistent with volcanic degassing and ventilation-related processes acting as strong deterministic filters during early colonization. This association suggests that the main compositional gradient among samples covaries with CO_2_ source/mixing dynamics and air exchange, integrating key stressors such as gas inputs and ventilation regimes. In contrast, at the genus level no measured environmental or mineralogical variable showed a significant association with the ordination (Fig. 8B; *p* ≥ 0.05), indicating that finer-scale taxonomic turnover is not structured by a single dominant factor but likely reflects the combined influence of multiple microhabitat constraints and sample-specific contingencies. Together, these patterns are consistent with broad stochastic seeding followed by deterministic filtering, where ventilation/degassing signals captured by *δ*^13^C-CO_2_ act as system-level constraints while local mineralogical heterogeneity contributes to within-system compositional divergence.

The co-occurrence network (Fig. 9) provides complementary evidence that microbial community structure reflects localized associations among taxa, minerals, and microenvironmental variables, rather than a single dominant driver at the genus level. Several highly connected taxa emerge as putative keystone nodes, including *Arthrobacter* (phylum *Actinomycetota*), *Anaerococcus* and *Bacillus* (phylum *Bacillota*), *Pseudomonas* (phylum *Pseudomonadota*), and *Nocardioides* (phylum *Actinomycetota*). Their high betweenness centrality and extensive positive associations suggest that these genera may act as connectors across redox-active microhabitats and mineral surfaces, consistent with observations from other volcanic cave systems [10, 88]. Together, these bacterial phyla appear to constitute the metabolic backbone of the lava tube microbiome, supporting processes such as organic matter turnover, biomineralization, and tolerance to oligotrophic basaltic substrates. Additional mineral-associated modules, particularly those linking heterotrophic facultative taxa (*Pedobacter*, *Atopostipes*, *Clostridium*) with evaporitic minerals (e.g., aphthitalite, augite) and *δ*^13^C-CO_2_, further suggests coupling between carbon cycling and mineral surface chemistry [54]. Importantly, these network patterns should be interpreted as taxon-level co-variation signals, which can capture microhabitat-scale structuring even when environmental fitting does not yield significant associations with the global genus-level ordination (Fig. 8B). In line with this, Pearson correlations (Table S3) indicate that diversity covaries with methane-related parameters (positive with CH_4_ concentration and negative with *δ*^13^C-CH_4_), consistent with more reduced, methane-influenced conditions.

Community assembly metrics further support a mixed scenario where stochastic inputs interact with strong microhabitat constraints. Near-zero NTI values across samples (Fig. 10) are consistent with an important stochastic component during early establishment (e.g., chance arrival and priority effects). A limited fit of Sloan’s neutral model indicates that neutral dynamics alone cannot fully explain abundance-occupancy patterns, and taxa deviating from neutral predictions likely reflect selective persistence under pronounced physicochemical heterogeneity (temperature-humidity contrasts and Na-rich saline mineral assemblages). Together, these results align with stochastic seeding (atmospheric and animal-mediated inputs) followed by microhabitat-scale environmental filtering, resulting in spatially heterogeneous communities. Overall, the combined ordination, network and assembly analyses suggest that keystone bacterial taxa (*Bacillota*, *Actinomycetota*, and *Pseudomonadota*) underpin early community connectivity, while archaeal lineages, particularly halophilic *Methanobacteriota* (e.g., *Halostagnicola*, *Halonotius* and *Halorubrum*), point to microhabitat filtering in these newly formed lava tubes.

## Conclusions

This study provides the first geomicrobiological investigation of the newly formed lava tubes generated by the 2021 Tajogaite eruption, offering new insights into microbial colonization during the earliest stages of subterranean ecosystem development.

Pioneer microbial communities were dominated by bacterial lineages (*Bacillota*, *Actinomycetota* and *Pseudomonadota*) together with halophilic *Methanobacteriota*, consistent with strong environmental filtering in Na-rich, highly soluble sulfate-carbonate mineral microhabitats. Principal coordinates analyses with environmental fitting further linked phylum-level community turnover to *δ*^13^C-CO_2_, suggesting that volcanic degassing and ventilation-related processes contribute to deterministic constraints during early assembly.

In parallel, the detection of host-associated and opportunistic taxa (e.g., *Staphylococcus*, *Sphingomonas*), and isolates previously reported from mammalian feces (*Psychrobacillus vulpis*, *Filibacter tadaridae*), and nematode-associated phytopathogens (*Rathayibacter festucae*), indicate that external organic inputs can seed and contribute to early community assembly. Field evidence of bird guano, feathers, and nesting material near lava tube entrances, supports the possibility that localized nutrient hotspots facilitate the establishment of heterotrophic communities.

Altogether, our findings support a dual assembly scenario in which stochastic seeding (via aerosols and animal-mediated inputs) is followed by deterministic filtering imposed by degassing/ventilation signals and extreme geochemical conditions, yielding spatially heterogeneous communities across the newly formed lava tubes. These subterranean ecosystems therefore represent a unique natural laboratory for understanding primary succession, microbe-mineral interactions and early ecosystem assembly in basaltic lava tubes.

## Data Availability

The sequence data generated and analysed in this study are available under NCBI BioProject ID PRJNA1167275 and accession numbers PP902169-PP902183.
